# Supplementary material for: Are values stable throughout adulthood? Evidence from two German long-term panel studies
Source: PLoS One. 2023 Nov 30;18(11):e0289487. doi: 10.1371/journal.pone.0289487 (PMC10688669; doi:10.1371/journal.pone.0289487)
Supplement: S1 File — (DOCX) [file pone.0289487.s001.docx]

**Supplementary Materials**

# Value Instruments

Kluckhohn Questionnaire [from GSOEP](https://paneldata.org/soep-core/inst/soep-core-2016-pe/6)

The items are based (albeit with some changes of wording) on a classification of goals and measures initially developed by [1] and translated into German by [2]. More information on the development of the scale is given by [3]. Below we list the instructions and each item with the original domains in parentheses (S = success, F = Family Life, A = Altruism), our own assessment of the relevant Schwartz value in curly brackets and the corresponding Schwartz values as categorized by Shalom Schwartz in private correspondence in brackets. The English translation is shown first, followed by the German version (original).

Different things are important to different people, how important are the following things to you? Scale: 1 very important/ 2 important/ 3 less important/ 4 unimportant

1. to be able to afford something. (S) {PO} [SD/PO]
2. to help others (A) {BE} [BE/UN]
3. to fulfill one's potential. (S) {AC} [UN/SD]
4. to have success in the job (S) {AC} [AC/PO]
5. to have an own house (F) {PO/SE} [SE/PO/AC/SD]
6. to have a happy marriage F) {TR} [SE/BE/CO]
7. to have children (F) {CO} [BE/SE/CO]
8. to be socially and politically active (A) {SD/UN} [ALL EXCEPT CO/SE/HE]
9. to be able to travel and see the world. (S) {ST/SD} [ST/SD/UN/HE]

Note: In some waves of the GSOEP this questionnaire also includes an item asking about “Faith, religion” and “Spending a lot of time with friends”. These items were not asked in the commissioned questionnaire and therefore excluded from analysis.

**References**

[1] Kluckhohn, F. R., & Strodtbeck, F. L. (1961). *Variations in value orientations*. Evanston, IL: Row, Peterson.

[2] Bielenski, H. & Strümpel, B. (1988). Eingeschränkte Erwerbsarbeit bei Frauen und Männern. Fakten - Wünsche – Realisierungschancen [Restricted gainful employment among women and men. Facts - wishes - chances of realization]. Edition Sigma.

[3] Headey, B. (2008). Life goals matter to happiness: A revision of set point theory. *Social Indicators Research, 86,* 213-231. <https://doi.org/10.1007/s11205-007-9138-y>

## German Version

Verschiedenen Menschen sind verschiedene Dinge wichtig. Sind für Sie persönlich die folgenden Dinge heute ... sehr wichtig, wichtig, weniger wichtig oder ganz unwichtig?

1 Sehr wichtig/2 Wichtig /3 Weniger wichtig /4 Ganz unwichtig

1. Sich etwas leisten können
2. Für andere da sein
3. Sich selbst verwirklichen
4. Erfolg im Beruf haben
5. Ein eigenes Haus haben
6. Eine glückliche Ehe/Partnerschaft haben
7. Kinder haben
8. Sich politisch, gesellschaftlich einsetzen
9. Die Welt sehen, viele Reisen machen
10. Der Glaube, die Religion

## PVQ21

Scale: 1 not like me at all /2 not like me /3 a little like me /4 somewhat like me /5 like me /6 very much like me. Values: CO = conformity, TR = Tradition, BE = Benevolence, UN = Universalism, SD = Self-direction, ST = Stimulation, HE = Hedonism, AC = Achievement, PO = Power, SC= Security

**Table A**

Starting matrix of PVQ21 items

| Value Item |  | y |
| --- | --- | --- |
| SD1 | .50 | .87 |
| PO1 | .50 | -.87 |
| UN1 | -.71 | .71 |
| AC1 | .71 | -.71 |
| SC1 | -.50 | -.87 |
| ST1 | .87 | .50 |
| CO1 | -.87 | -.50 |
| UN2 | -.71 | .71 |
| TR1 | -.71 | -.71 |
| HE1 | 1 | 0 |
| SD2 | .50 | .87 |
| BE1 | -.87 | .50 |
| AC2 | .71 | -.71 |
| SC2 | -.50 | -.87 |
| ST2 | .87 | .50 |
| CO2 | -.87 | -.50 |
| PO2 | .50 | -.87 |
| BE2 | -.87 | .50 |
| UN3 | -.71 | .71 |
| TR2 | -.71 | - .71 |
| HE1 | 1 | 0 |

1. Thinking up new ideas and being creative is important to him. He likes to do things in his own original way (SD1)
2. It is important to him to be rich. He wants to have a lot of money and expensive things. (PO1)
3. He thinks it is important that every person in the world should be treated equally. He believes everyone should have equal opportunities in life. (UN1)
4. It’s important to him to show his abilities. He wants people to admire what he does. (AC1)
5. It is important to him to live in secure surroundings. He avoids anything that might endanger his safety (SC1)
6. He likes surprises and is always looking for new things to do. He thinks it is important to do lots of different things in life. (ST1)
7. He believes that people should do what they’re told. He thinks people should follow rules at all times, even when no one is watching. (CO1)
8. It is important to him to listen to people who are different from him. Even when he disagrees with them, he still wants to understand them. (UN2)
9. It is important to him to be humble and modest. He tries not to draw attention to himself. (TR1)
10. Having a good time is important to him. He likes to “spoil” himself. (HE1)
11. It is important to him to make his own decisions about what he does. He likes to be free and not depend on others. (SD2)
12. It’s very important to him to help the people around him. He wants to care for their well-being. (BE1)
13. Being very successful is important to him. He hopes people will recognize his achievements. (AC2)
14. It is important to him that the government ensures his safety against all threats. He wants the state to be strong so it can defend its citizens. (SC2)
15. He looks for adventures and likes to take risks. He wants to have an exciting life. (ST2)
16. It is important to him always to behave properly. He wants to avoid doing anything people would say is wrong. (CO2)
17. It is important to him to get respect from others. He wants people to do what he says. (PO2)
18. It is important to him to be loyal to his friends. He wants to devote himself to people close to him. (BE2)
19. He strongly believes that people should care for nature. Looking after the environment is important to him. (UN3)
20. Tradition is important to him. He tries to follow the customs handed down by his religion or his family (TR2)
21. He seeks every chance he can to have fun. It is important to him to do things that give him pleasure. (HE2)

### German Version

Im Folgenden beschreibe ich Ihnen einige Personen. Bitte benutzen Sie Liste 69 und sagen
Sie mir, wie ähnlich oder unähnlich Ihnen die jeweils beschriebene Person ist.

Response scale: 1 Ist mir sehr ähnlich\ 2 Ist mir ähnlich \3 Ist mir etwas ähnlich \4 Ist mir nur ein kleines bisschen ähnlich\ 5 Ist mir nicht ähnlich\ 6 Ist mir überhaupt nicht ähnlich

1. Es ist ihm wichtig, neue Ideen zu entwickeln und kreativ zu sein. Er macht Sache gerne auf seine eigene originelle Art und Weise.
2. Es ist ihm wichtig, reich zu sein. Er möchte viel Geld haben und teure Sachen besitzen
3. Er hält es für wichtig, dass alle Menschen auf der Welt gleich behandelt werden sollten Er glaubt, dass jeder Mensch im Leben gleiche Chancen haben sollte.
4. Es ist ihm wichtig, seine Fähigkeiten zu zeigen. Er möchte, dass die Leute bewundern, was er tut.
5. Es ist ihm wichtig, in einem sicheren Umfeld zu leben. Er vermeidet alles, was seine Sicherheit gefährden könnte.
6. Er mag Überraschungen und hält immer Ausschau nach neuen Aktivitäten. Er denkt, dass im Leben Abwechslung wichtig ist.
7. Er glaubt, dass die Menschen tun sollten, was man ihnen sagt. Er denkt, dass Menschen sich immer an Regeln halten sollten, selbst dann, wenn es niemand sieht
8. Es ist ihm wichtig, Menschen zuzuhören, die anders sind als er. Auch wenn er anderer Meinung ist als andere, will er die anderen trotzdem verstehen.
9. Es ist ihm wichtig, zurückhaltend und bescheiden zu sein. Er versucht, die Aufmerksamkeit nicht auf sich zu lenken.
10. Es ist ihm wichtig, Spaß zu haben. Er gönnt sich selbst gerne etwas.
11. Es ist ihm wichtig, selbst zu entscheiden, was er tut. Er ist gerne frei und unabhängig von anderen.
12. Es ist ihm sehr wichtig, den Menschen um ihn herum zu helfen. Er will für deren Wohl sorgen
13. Es ist ihm wichtig, sehr erfolgreich zu sein. Er hofft, dass die Leute seine Leistungen anerkennen.
14. Es ist ihm wichtig, dass der Staat seine persönliche Sicherheit vor allen Bedrohungen gewährleistet. Er will einen starken Staat, der seine Bürger verteidigt.
15. Er sucht das Abenteuer und geht gerne Risiken ein. Er will ein aufregendes Leben haben.
16. Es ist ihm wichtig, sich jederzeit korrekt zu verhalten. Er vermeidet es, Dinge zu tun, die andere Leute für falsch halten könnten
17. Es ist ihm wichtig, dass andere ihn respektieren. Er will, dass die Leute tun, was er sagt.
18. Es ist ihm wichtig, seinen Freunden gegenüber loyal zu sein. Er will sich für Menschen einsetzen, die ihm nahestehen
19. Er ist fest davon überzeugt, dass die Menschen sich um die Natur kümmern sollten. Umweltschutz ist ihm wichtig.
20. Tradition ist ihm wichtig. Er versucht, sich an die Sitten und Gebräuche zu halten, die ihm von seiner Religion oder seiner Familie überliefert wurden
21. Er lässt keine Gelegenheit aus, Spaß zu haben. Es ist ihm wichtig, Dinge zu tun, die ihm Vergnügen bereiten.

## SVS-10 from Life under Nuclear Threat Dataset

The SVS-10 items and instructions are reported here in English and German (bold) with starting matrix for the weak confirmatory MDS on the right.

“Below you can find 10 values that can be important to people. Please mark for each of these values how important it is for you.” Scale: -1 contrary to the value of the concerned person, 0 not important to 7 very important

**Table B**

**Starting Matrix of SVS-10 Items**

| Item | X | Y |
| --- | --- | --- |
| helpful/**Hilfsbereitschaft** (BE) | -.87, | .50 |
| social order/**Soziale Ordnung** (SC) | -.50, | -.87 |
| success/**Erfolg** (AC) | .71, | -.71 |
| protection of the Environment/**Umweltschutz** (UN) | -.71, | .71 |
| polite/**Höflichkeit** (CO) | -.87, | -.50 |
| creative/**Kreativität** (SD) | .50, | .87 |
| pleasure/**Vergnügen** (HE) | 1,00 | 0 |
| respect for tradition/**Achtung** **vor der Tradition** (TR) | .71, | -.71 |
| social power/**Soziale** **Macht** (PO) | .50, | -.87 |
| daring/**Wagemut** (ST) | .87, | .50 |

# MDS of Kluckhohn-Strodtbeck Items

| **Fig A**  **Best Kluckhohn-Strodtbeck MDS Solution** |
| --- |
| 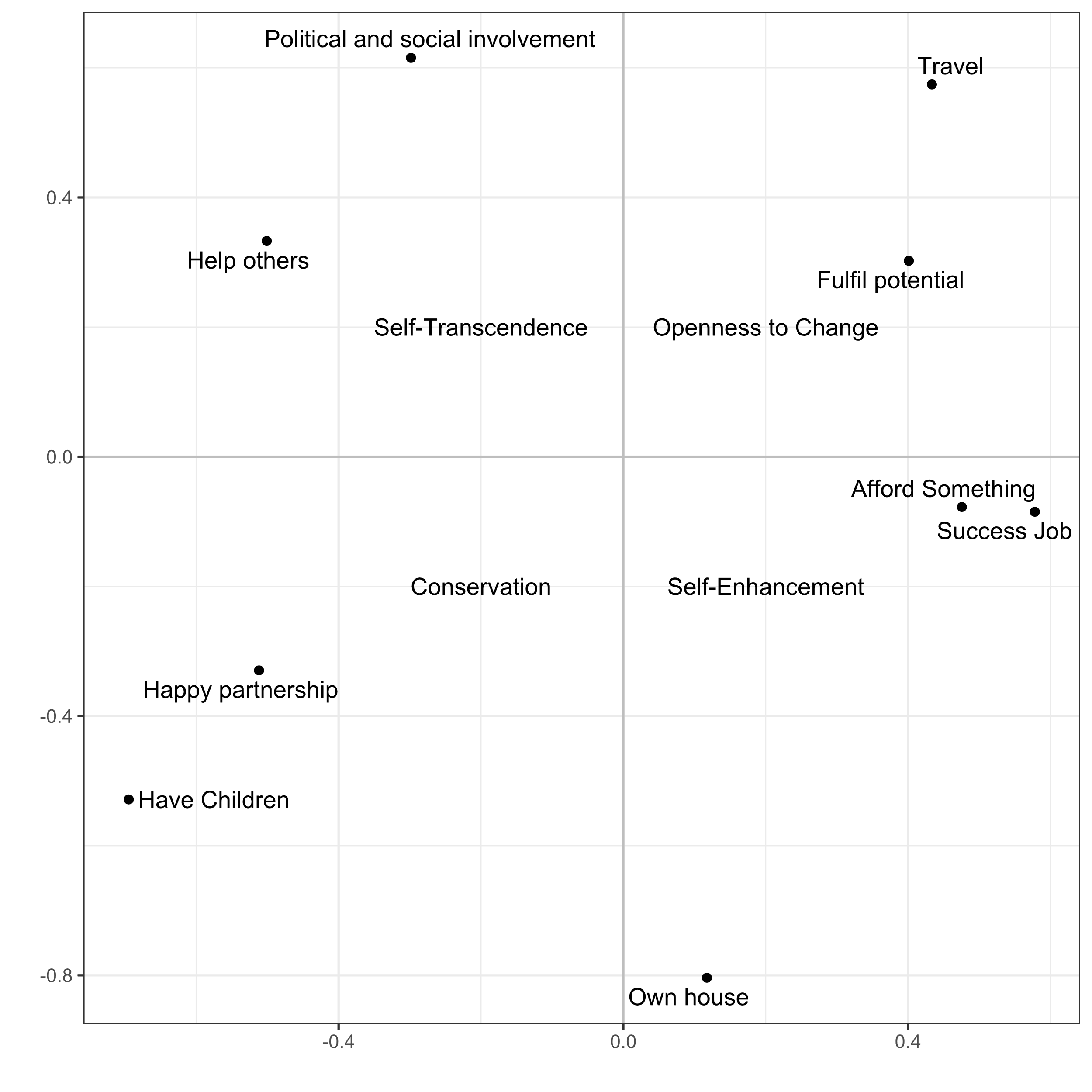  *Note.* Using ipsatized items from 1000 random starting locations with quadrants labelled with proposed higher-order value orientations |

| **Table C**  **Correlation Matrix of Kluckhohn Ipsatized Items in Study 1** | | | | | | | | |
| --- | --- | --- | --- | --- | --- | --- | --- | --- |
|  | Afford | Help others | Fulfil potential | Job success | Own house | Happy partnership | Have children | Political and social involvement |
| Help others | -.15 |  |  |  |  |  |  |  |
| Fulfil potential | .04 | -.07 |  |  |  |  |  |  |
| Job success | .10 | -.18 | .06 |  |  |  |  |  |
| Own house | -.11 | -.32 | -.25 | -.05 |  |  |  |  |
| Happy partnership | -.08 | .02 | -.19 | -.22 | -.06 |  |  |  |
| Have children | -.31 | -.03 | -.36 | -.24 | -.01 | .04 |  |  |
| Political and social involvement | -.20 | .08 | -.01 | -.15 | -.21 | -.20 | -.23 |  |
| Travel and see the world | .01 | -.17 | .04 | -.13 | -.22 | -.21 | -.27 | -.08 |

| **Fig B**  **Positions of Kluckhohn-Strodtbeck Items with 95% Confidence Intervals** |
| --- |
| 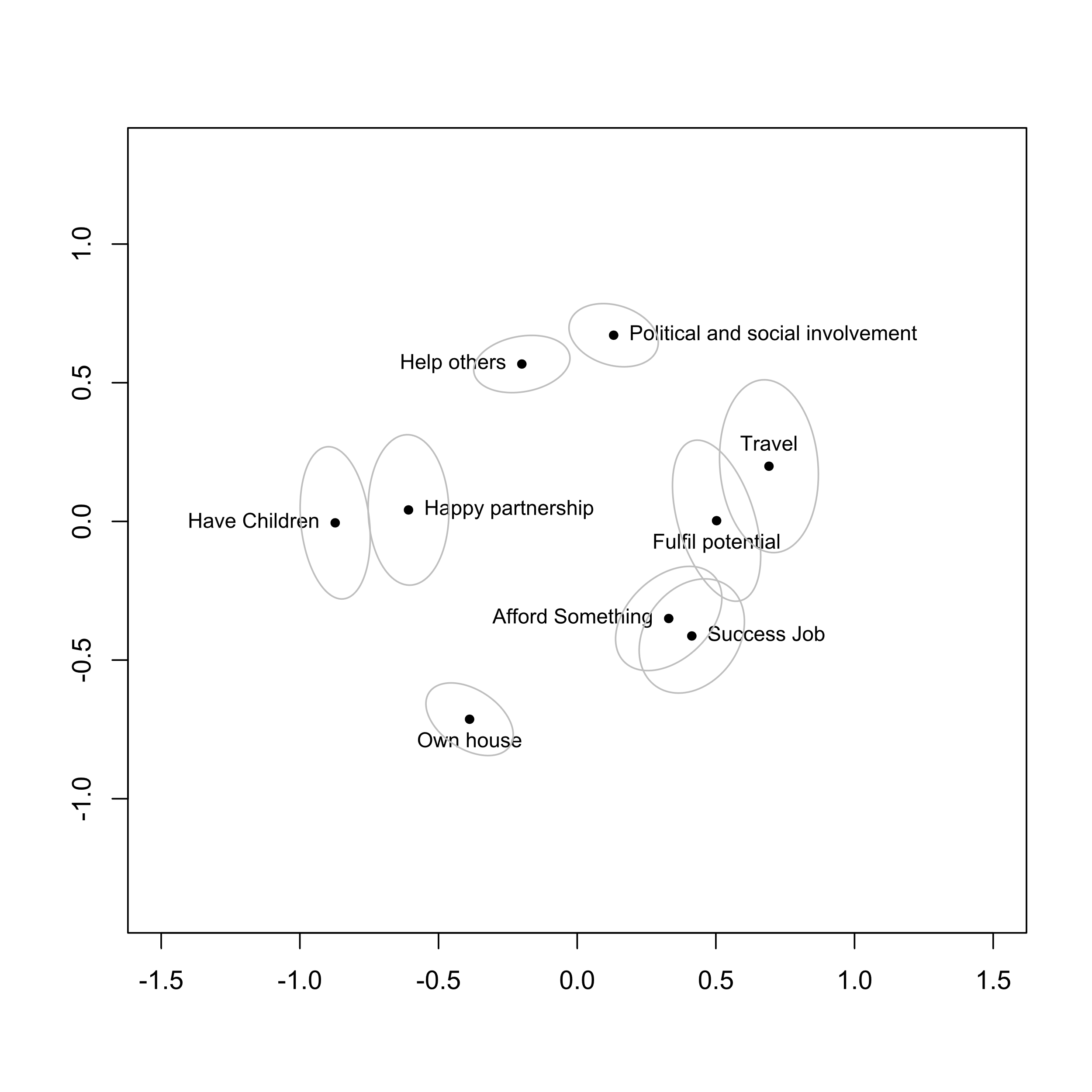  *Note.* From 1000 random starts MDS |
|  |

| **Fig C**  **Positions of Kluckhohn-Strodtbeck Items** |
| --- |
| 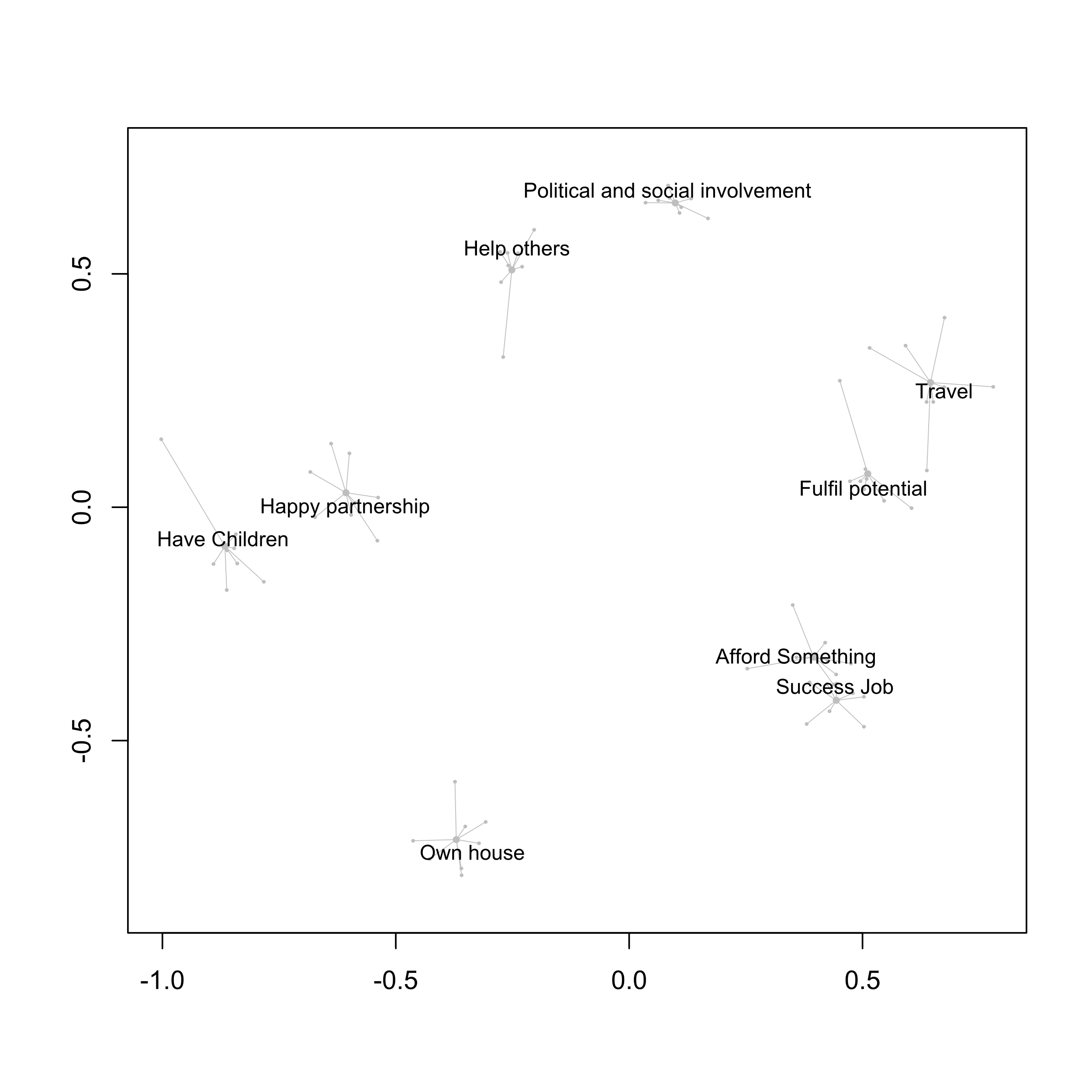  *Note.* With alternative position from jackknife procedure in grey |

# MDS Solution of the SVS-10 Items in Study 1

| **Figure D**  **Confirmatory MDS of SVS10 Items** |
| --- |
| 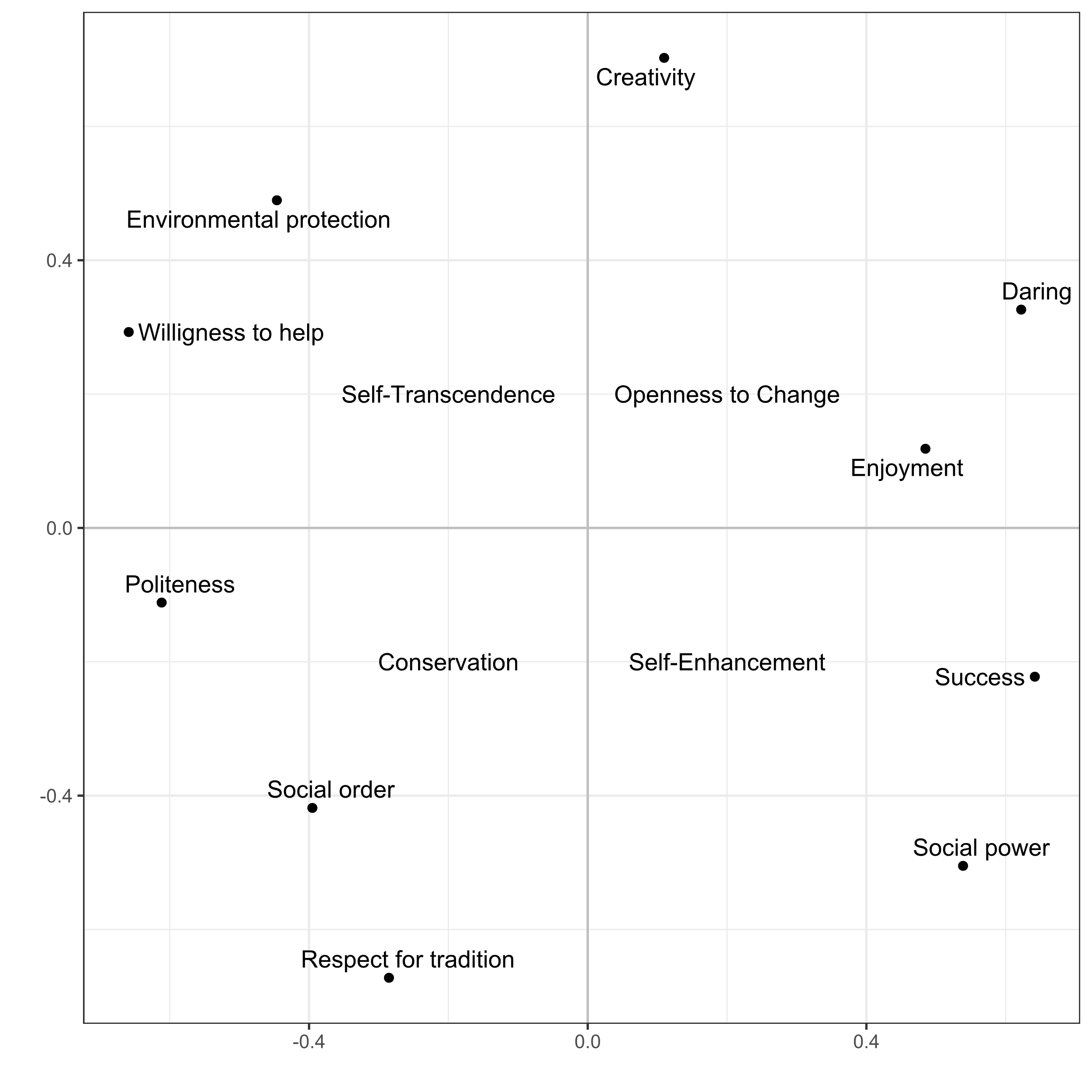 |

*Note*. With quadrants labelled with proposed higher-order value orientations

| **Table D**  **Correlation Matrix of SVS Ipsatized Items Study 1** | | | | | | | | | |
| --- | --- | --- | --- | --- | --- | --- | --- | --- | --- |
|  | BE | UN | SD | ST | HE | AC | PO | SE | TR |
| UN | .11 |  |  |  |  |  |  |  |  |
| SD | -.04 | -.01 |  |  |  |  |  |  |  |
| ST | -.18 | -.13 | .00 |  |  |  |  |  |  |
| HE | -.18 | -.17 | -.11 | .04 |  |  |  |  |  |
| AC | -.28 | -.25 | -.12 | -.03 | .04 |  |  |  |  |
| PO | -.33 | -.26 | -.18 | .03 | -.10 | .10 |  |  |  |
| SE | -.05 | -.09 | -.21 | -.30 | -.16 | -.15 | -.06 |  |  |
| TR | -.14 | -.22 | -.20 | -.22 | -.21 | -.13 | -.10 | .04 |  |
| CO | .35 | -.03 | -.21 | -.27 | -.03 | -.22 | -.31 | .06 | .00 |

| **Fig E**  **Positions of SVS-10 Items with 95% Confidence Intervals from Confirmatory MDS** |
| --- |
| 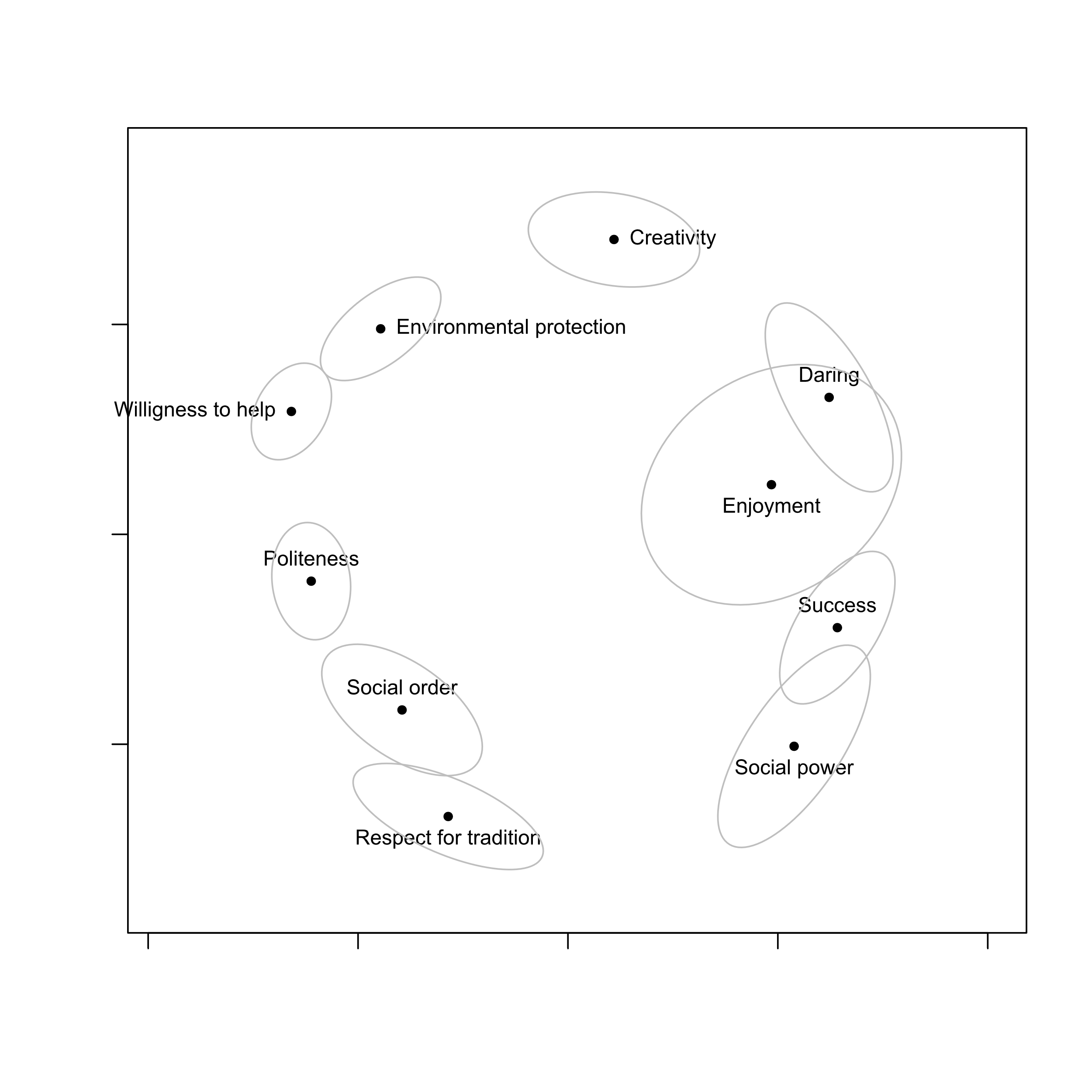 |

| **Fig F**  **Positions of SVS-10 Items** |
| --- |
| 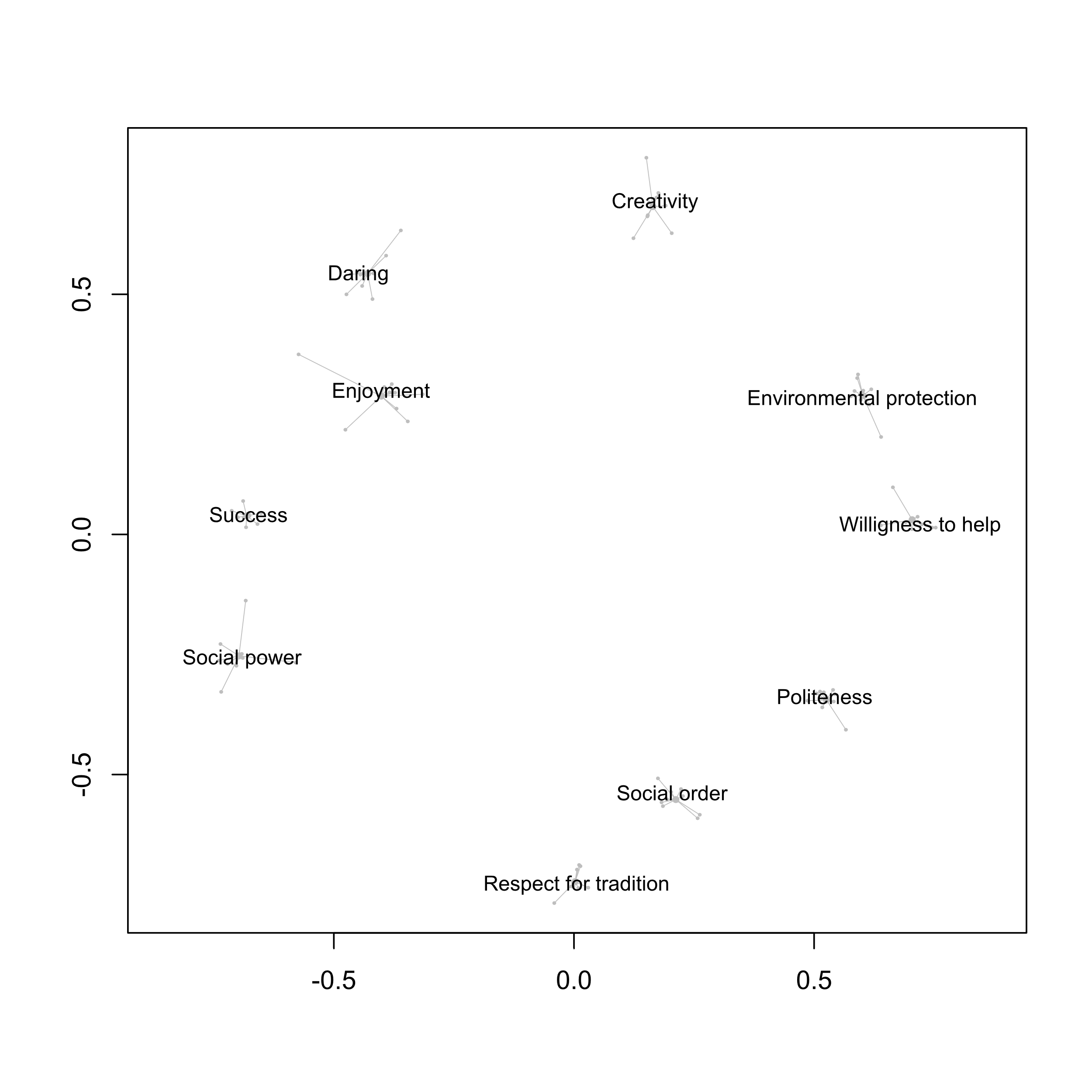  *Note* With alternative position from jackknife procedure in grey. |

# MDS of PVQ-21

| **Fig G**  **Confirmatory MDS of Ipsatized PVQ-21 Items** |
| --- |
| 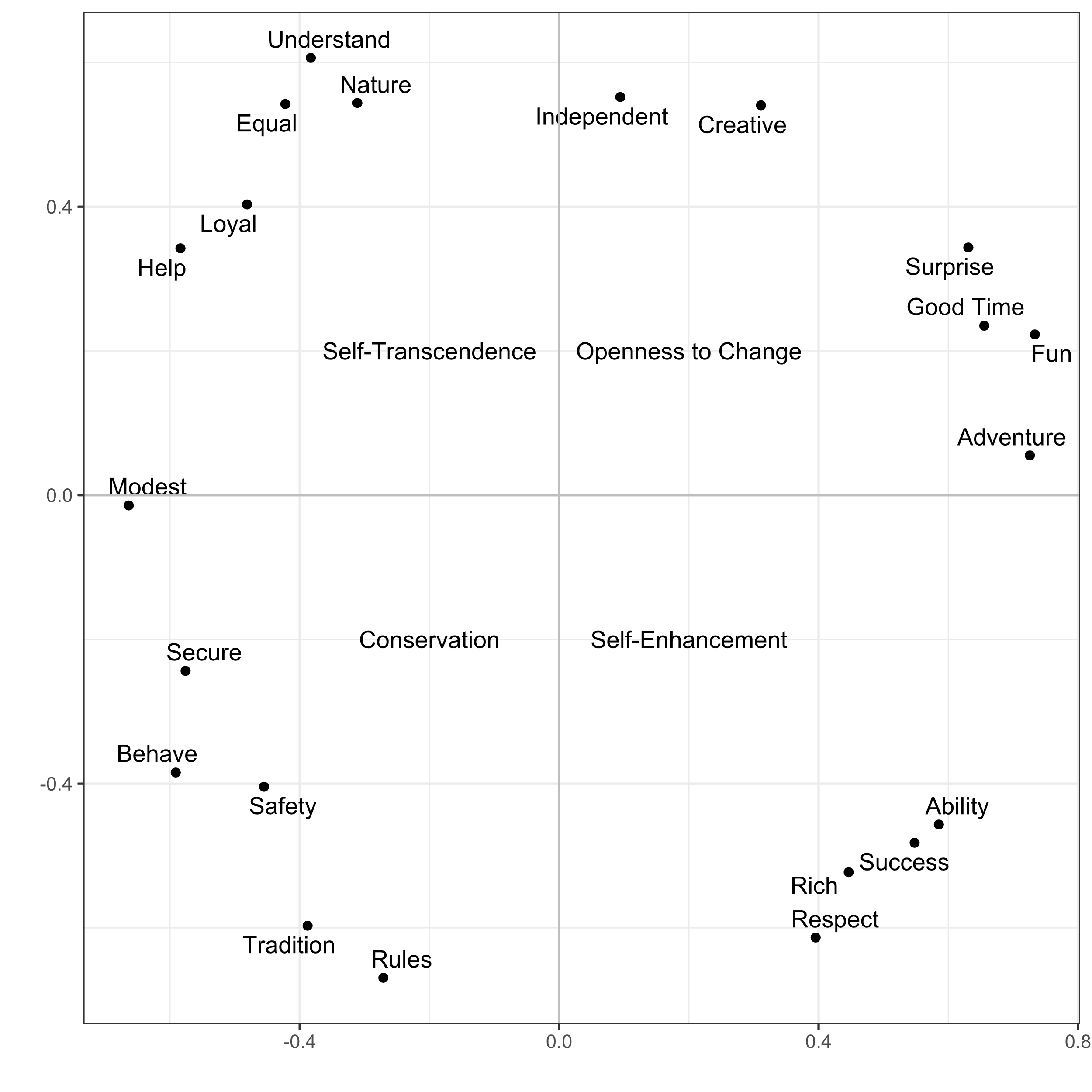 |

*Note.* With quadrants labelled with the proposed higher-order value orientations

| **Fig H**  **Bootstrapped MDS of Ipsatized PVQ-21 Items** |
| --- |
| 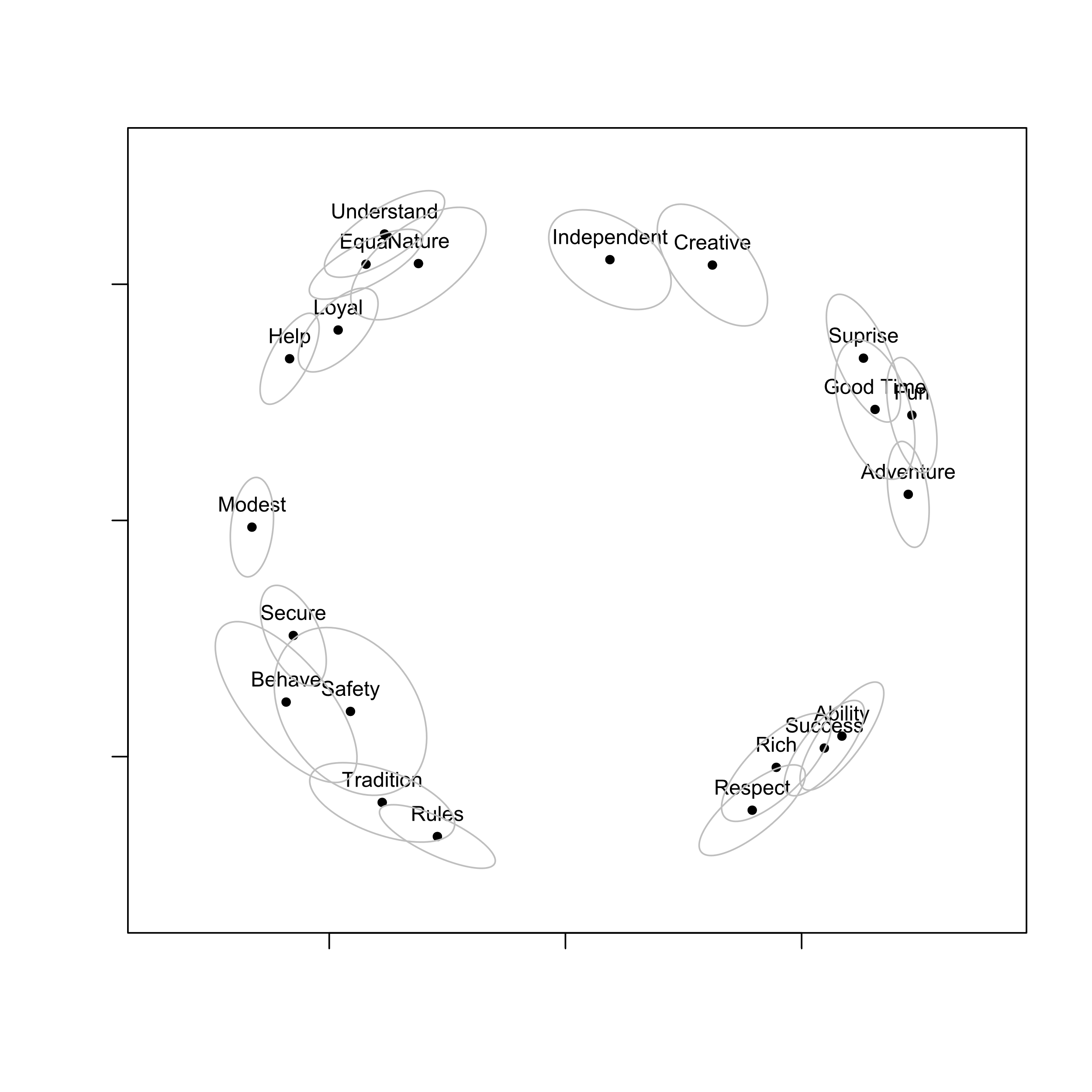 |

| **Fig I**  **Jackknife MDS of Ipsatized PVQ-21 Items** |
| --- |
| 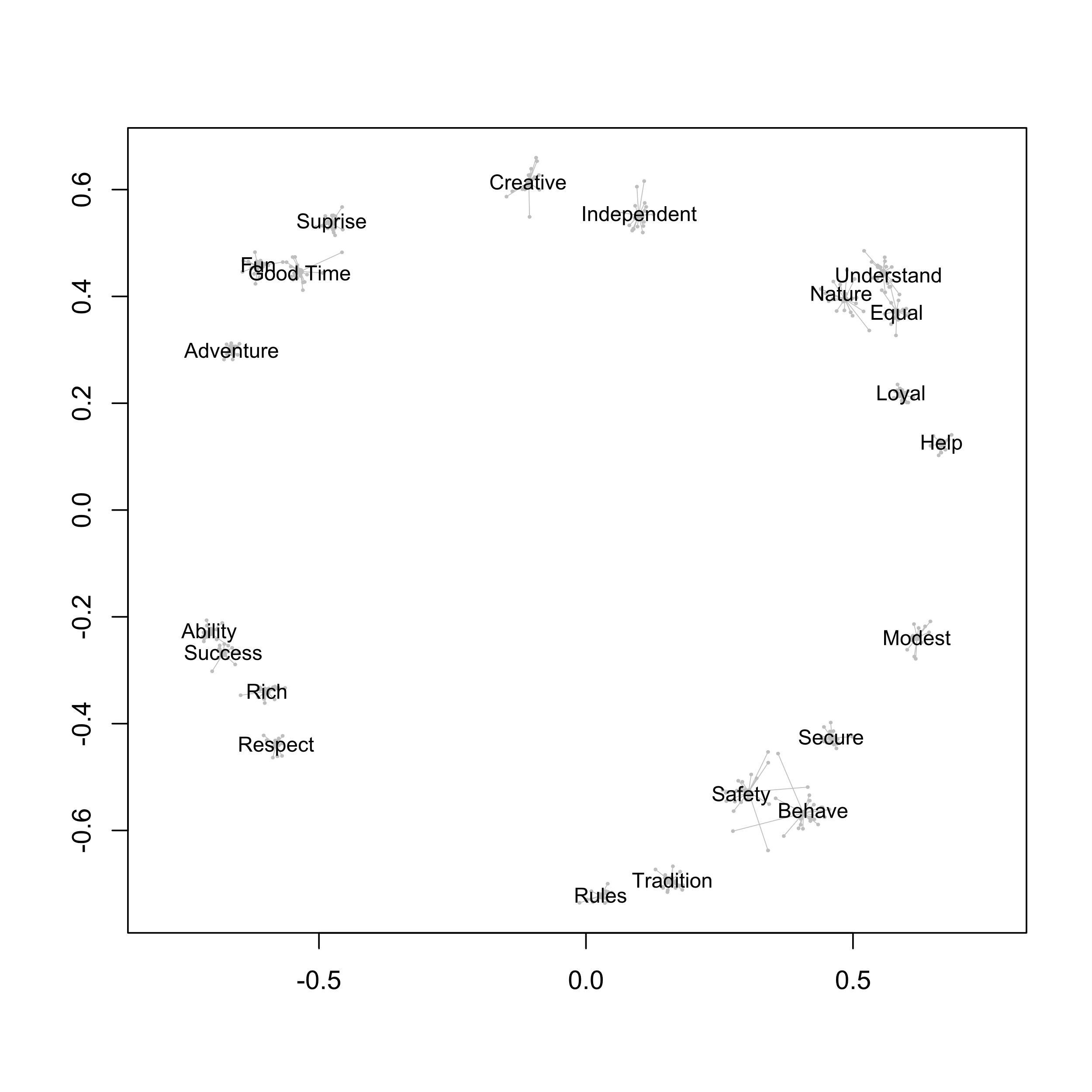 |

| **Table E**  **Correlation Matrix of Ipsatized PVQ-21 Items in Study 1** | | | | | | | | | | | | | | | | | | | | |
| --- | --- | --- | --- | --- | --- | --- | --- | --- | --- | --- | --- | --- | --- | --- | --- | --- | --- | --- | --- | --- |
|  | SD1 | PO1 | UN1 | AC1 | SE1 | ST1 | CO1 | UN2 | TR1 | HE1 | SD2 | BE1 | AC2 | SE2 | ST2 | CO2 | PO2 | BE2 | UN3 | TR2 |
| PO1 | -.08 |  |  |  |  |  |  |  |  |  |  |  |  |  |  |  |  |  |  |  |
| UN1 | .02 | -.35 |  |  |  |  |  |  |  |  |  |  |  |  |  |  |  |  |  |  |
| AC1 | -.01 | .24 | -.21 |  |  |  |  |  |  |  |  |  |  |  |  |  |  |  |  |  |
| SE1 | -.25 | -.04 | -.06 | -.16 |  |  |  |  |  |  |  |  |  |  |  |  |  |  |  |  |
| ST1 | .18 | -.02 | -.06 | .00 | -.40 |  |  |  |  |  |  |  |  |  |  |  |  |  |  |  |
| CO1 | -.23 | .04 | -.17 | -.05 | .14 | -.29 |  |  |  |  |  |  |  |  |  |  |  |  |  |  |
| UN2 | .10 | -.31 | .36 | -.19 | -.11 | -.07 | -.21 |  |  |  |  |  |  |  |  |  |  |  |  |  |
| TR1 | -.15 | -.23 | .20 | -.32 | .22 | -.27 | .12 | .11 |  |  |  |  |  |  |  |  |  |  |  |  |
| HE1 | -.04 | .01 | -.09 | .01 | -.17 | .21 | -.23 | -.09 | -.24 |  |  |  |  |  |  |  |  |  |  |  |
| SD2 | .09 | -.11 | .10 | -.15 | -.02 | .04 | -.25 | .04 | -.06 | .10 |  |  |  |  |  |  |  |  |  |  |
| BE1 | -.01 | -.35 | .22 | -.25 | .08 | -.14 | -.09 | .33 | .14 | -.17 | -.06 |  |  |  |  |  |  |  |  |  |
| AC2 | .00 | .33 | -.25 | .36 | -.17 | .03 | -.06 | -.21 | -.30 | -.06 | -.16 | -.20 |  |  |  |  |  |  |  |  |
| SE2 | -.15 | -.05 | -.16 | -.20 | .32 | -.25 | .14 | -.09 | .06 | -.12 | .00 | -.02 | -.16 |  |  |  |  |  |  |  |
| ST2 | .10 | .11 | -.14 | .05 | -.42 | .50 | -.24 | -.17 | -.26 | .19 | -.01 | -.23 | .08 | -.26 |  |  |  |  |  |  |
| CO2 | -.28 | -.08 | .01 | -.07 | .27 | -.32 | .26 | -.08 | .26 | -.22 | -.24 | .01 | -.08 | .05 | -.35 |  |  |  |  |  |
| PO2 | -.10 | .27 | -.28 | .31 | -.07 | -.07 | .08 | -.24 | -.27 | -.06 | -.13 | -.24 | .23 | -.09 | .04 | -.04 |  |  |  |  |
| BE2 | -.03 | -.31 | .22 | -.27 | .06 | -.13 | -.17 | .25 | .07 | -.06 | .15 | .38 | -.28 | .02 | -.23 | .00 | -.22 |  |  |  |
| UN3 | .10 | -.28 | .25 | -.17 | -.01 | -.10 | -.12 | .19 | .04 | -.13 | .09 | .11 | -.20 | -.06 | -.11 | -.04 | -.19 | .15 |  |  |
| TR2 | -.14 | -.07 | -.21 | -.08 | .04 | -.17 | .22 | -.14 | .06 | -.21 | -.23 | .03 | -.02 | .16 | -.16 | .11 | -.06 | -.05 | -.15 |  |
| HE2 | -.04 | -.01 | -.14 | .00 | -.22 | .28 | -.16 | -.13 | -.23 | .46 | .06 | -.20 | -.06 | -.12 | .32 | -.26 | -.07 | -.14 | -.18 | -.17 |

# MDS of PVQ with HOV of SVS and K-S measurements

| **Fig J**  **Bootstrapped MDS of Ipsatized PVQ-21 Items and HOV from SVS-10 and K-S Instruments** |
| --- |
| 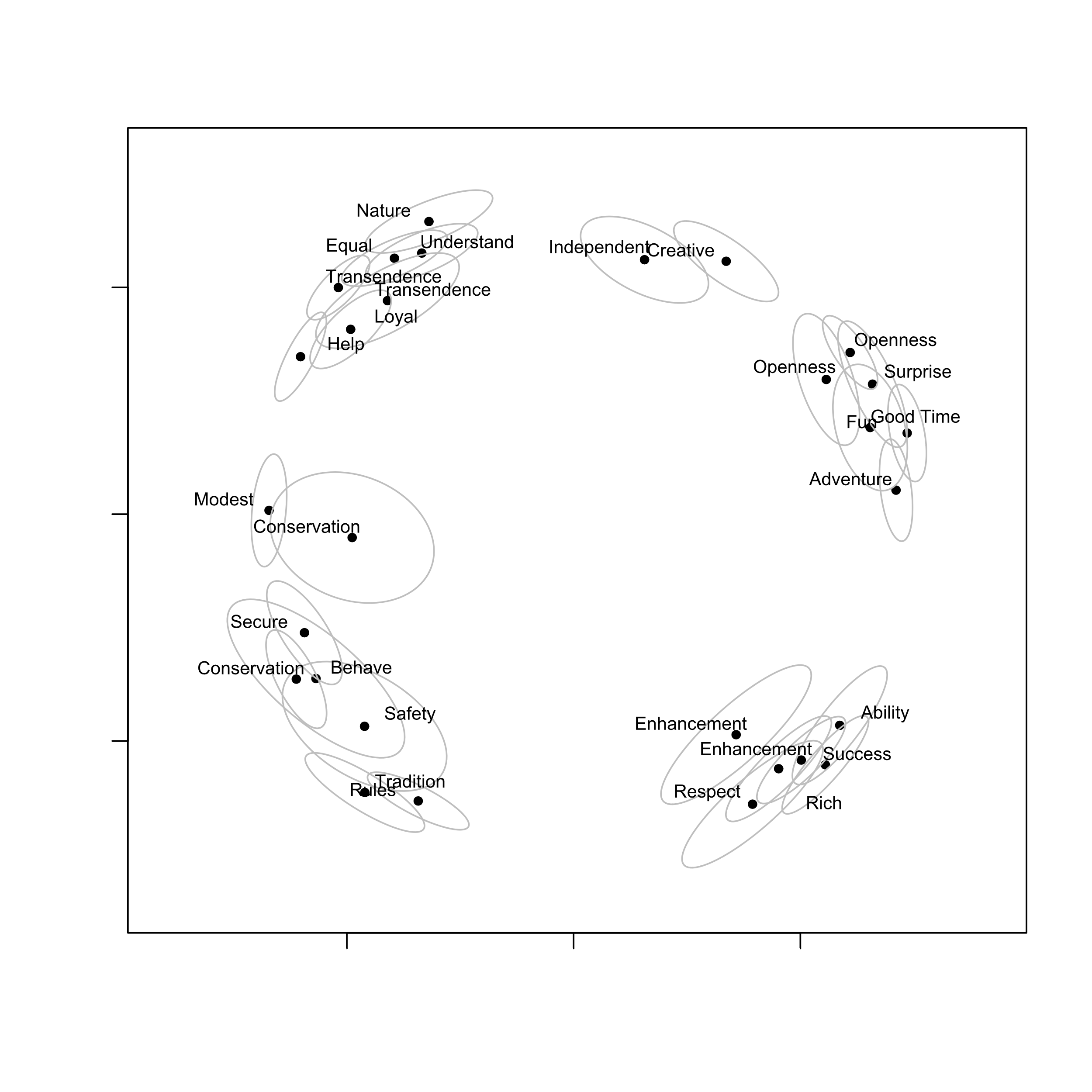 |

| **Fig K**  **Jackknife MDS of Ipsatized PVQ-21 Items and HOV from SVS-10 and K-S Instruments** | |
| --- | --- |
| 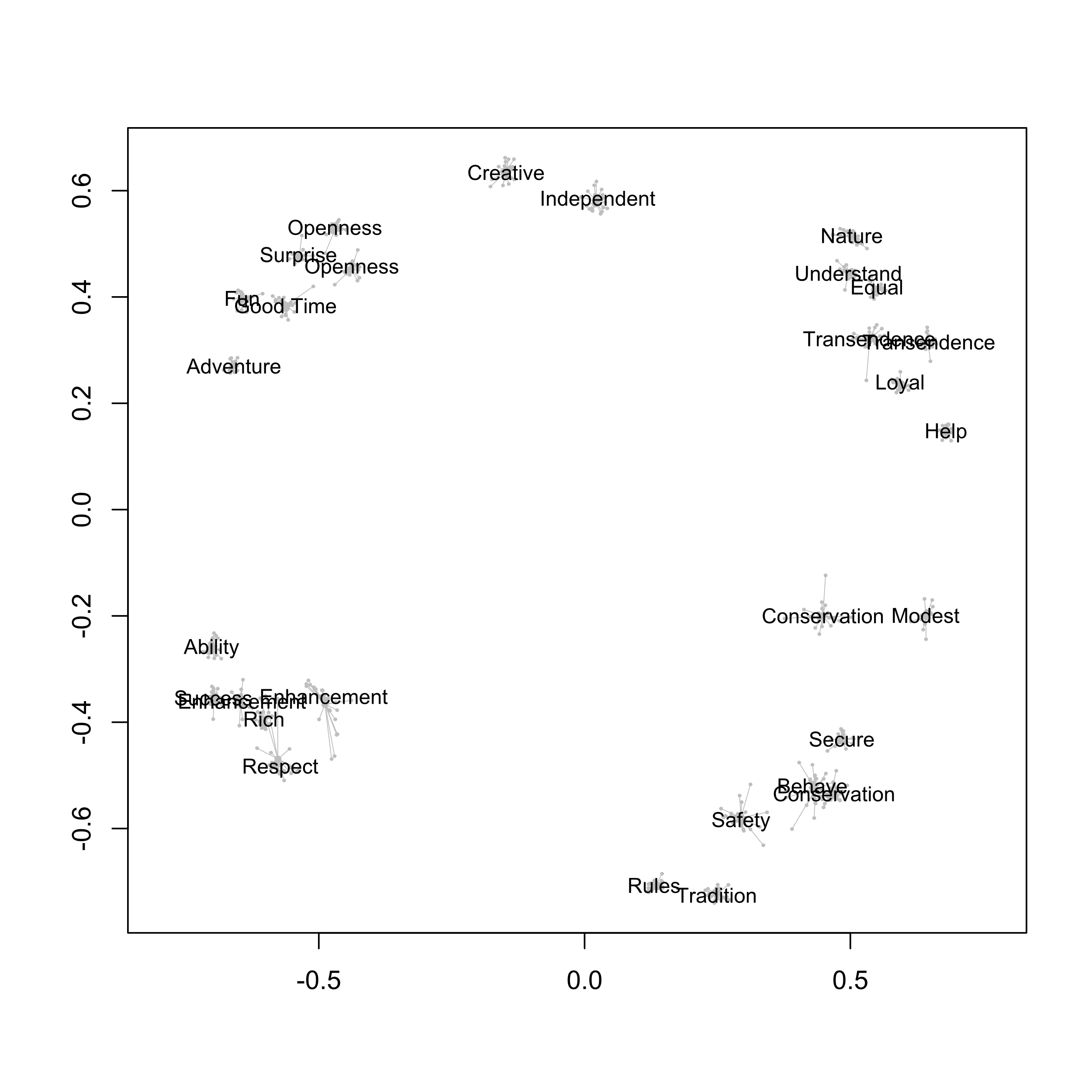 |  |

# Study 2 (LuNT) Descriptive Statistics

## Methods Information

All models follow the general formulation in Equation 1 which describes a multilevel model with fixed and random effects. In Equation 1, *i* refers to the *i^th^* observation of respondent *j*, $\beta_{0}$ is the population intercept, $\beta_{1}$ is the effect of one unit of time (year variable). $\beta_{2}$ refers to a fixed effect of year squared. Together, $\beta_{1}$and $\beta_{2}$ describe the average change in values between years. The term $u_{0j}$ refers to the random intercept and $u_{1j}year$ is the random effect of the variable *time* for each individual *j.* The random terms account for individual level ($u_{j}$) variation in the intercept ($\text{β}_{\text{0}}$) and year coefficient ($\beta_{1}$).

$$y_{ij}= \text{β}_{\text{0}}+\beta_{1}\mathrm{year}_{\mathrm{ij}}+\beta_{2}{\mathrm{year}^{2}}_{\mathrm{ij}}+u_{0j}+u_{1j}\mathrm{year}$$

**Table F**

**Descriptive Statistics of LuNT by Survey Year**

| Year | Variable | Mean | SD | min | max | Observations |
| --- | --- | --- | --- | --- | --- | --- |
| 1999 | Conservation | 4.64 | 1.29 | 0.00 | 7.67 | 234 |
| 2003 | Conservation | 4.75 | 1.28 | 1.67 | 8.00 | 197 |
| 2006 | Conservation | 4.86 | 1.17 | 1.33 | 7.67 | 193 |
| 2009 | Conservation | 4.88 | 1.25 | 1.00 | 8.00 | 204 |
| 2013 | Conservation | 5.09 | 1.27 | 2.00 | 8.00 | 188 |
| 2017 | Conservation | 5.07 | 1.24 | 2.00 | 7.67 | 191 |
| 1999 | Openness to Change | 4.74 | 1.42 | 1.00 | 8.00 | 235 |
| 2003 | Openness to Change | 4.63 | 1.45 | 1.00 | 8.00 | 197 |
| 2006 | Openness to Change | 4.59 | 1.36 | 1.00 | 8.00 | 193 |
| 2009 | Openness to Change | 4.57 | 1.47 | 1.00 | 8.00 | 204 |
| 2013 | Openness to Change | 4.64 | 1.53 | 1.00 | 8.00 | 188 |
| 2017 | Openness to Change | 4.70 | 1.55 | 1.50 | 8.00 | 191 |
| 1999 | Self-enhancement | 6.24 | 1.16 | 3.00 | 8.00 | 235 |
| 2003 | Self-enhancement | 6.17 | 1.19 | 1.00 | 8.00 | 197 |
| 2006 | Self-enhancement | 6.22 | 1.14 | 2.00 | 8.00 | 193 |
| 2009 | Self-enhancement | 6.29 | 1.09 | 2.00 | 8.00 | 204 |
| 2013 | Self-enhancement | 6.42 | 1.11 | 3.00 | 8.00 | 188 |
| 2017 | Self-enhancement | 6.40 | 1.10 | 2.50 | 8.00 | 192 |
| 1999 | Self-transcendence | 4.14 | 1.36 | 0.50 | 7.50 | 234 |
| 2003 | Self-transcendence | 4.08 | 1.33 | 1.00 | 8.00 | 197 |
| 2006 | Self-transcendence | 4.06 | 1.26 | 0.50 | 8.00 | 192 |
| 2009 | Self-transcendence | 4.01 | 1.44 | 1.00 | 8.00 | 204 |
| 2013 | Self-transcendence | 4.03 | 1.41 | 0.50 | 7.50 | 188 |
| 2017 | Self-transcendence | 3.90 | 1.45 | 0.00 | 7.50 | 191 |
| 1999 | age | 28.18 | 2.73 | 21.00 | 44.00 | 235 |
| 2003 | age | 32.18 | 2.78 | 25.00 | 48.00 | 197 |
| 2006 | age | 35.18 | 2.71 | 28.00 | 51.00 | 193 |
| 2009 | age | 38.25 | 2.79 | 31.00 | 54.00 | 204 |
| 2013 | age | 42.08 | 2.56 | 35.00 | 51.00 | 188 |
| 2017 | age | 46.25 | 2.79 | 39.00 | 62.00 | 192 |
| 1999 | Gender (% female) | 0.58 |  | 0.00 | 1.00 | 229 |
| 2003 | Gender (% female) | 0.60 |  | 0.00 | 1.00 | 191 |
| 2006 | Gender (% female) | 0.59 |  | 0.00 | 1.00 | 189 |
| 2009 | Gender (% female) | 0.58 |  | 0.00 | 1.00 | 200 |
| 2013 | Gender (% female) | 0.58 |  | 0.00 | 1.00 | 183 |
| 2017 | Gender (% female) | 0.59 |  | 0.00 | 1.00 | 188 |

# LuNT Model Fit Table

**Table G**

**Model Fit Statistics of Multilevel Growth Models Estimated on the LuNT HOV**

| Outcome | Fixed slopes | Random Slopes | LL | AIC | BIC | χ^2^ | χ^2^ *p* |
| --- | --- | --- | --- | --- | --- | --- | --- |
| Conservation |  |  | -1436 | 2878 | 2893 | n.a.. | n.a. |
| Conservation | linear |  | -1411 | 2830 | 2851 | 50 | <.01 |
| **Conservation** |  | **Linear** | **-1403** | **2818** | **2848** | **17** | **<.01** |
| Conservation | Squared | linear | -1402 | 2819 | 2854 | 1 | .26 |
| Conservation | Squared, cubic | linear | -1402 | 2820 | 2861 | 0 | .60 |
| Conservation |  | Linear, squared | -1399 | 2817 | 2868 | 7 | .06 |
| Conservation | cubic | Linear, squared | -1398 | 2819 | 2875 | 7 | .06 |
| Conservation |  | Linear, squared, cubic | -1396 | 2823 | 2899 | 4 | .37 |
| Openness to Change |  |  | -1435 | 2877 | 2892 | n.a.. | n.a. |
| Openness to Change | linear |  | -1435 | 2879 | 2899 | 0 | .92 |
| Openness to Change |  | Linear | -1429 | 2871 | 2901 | 12 | <.01 |
| **Openness to Change** | **Squared** | **linear** | **-1426** | **2865** | **2901** | **8** | **<.01** |
| Openness to Change | Squared, cubic | linear | -1426 | 2867 | 2908 | 0 | .71 |
| Openness to Change |  | Linear, squared | -1423 | 2866 | 2917 | 5 | .19 |
| Openness to Change | cubic | Linear, squared | -1423 | 2868 | 2924 | 5 | .19 |
| Self-Enhancement |  |  | -1444 | 2895 | 2910 | n.a. | n.a. |
| Self-Enhancement | linear |  | -1441 | 2890 | 2911 | 7 | .01 |
| **Self-Enhancement** |  | **Linear** | **-1432** | **2876** | **2906** | **19** | **<.01** |
| Self-Enhancement | Squared | linear | -1432 | 2877 | 2913 | 0 | .53 |
| Self-Enhancement | Squared, cubic | linear | -1431 | 2879 | 2919 | 1 | .38 |
| Self-Enhancement |  | Linear, squared | -1430 | 2880 | 2931 | 4 | .28 |
| Self-Enhancement | cubic | Linear, squared | -1429 | 2881 | 2937 | 4 | .28 |
| Self-Enhancement |  | Linear, squared, cubic | -1426 | 2883 | 2959 | 6 | .21 |
| Self-Transcendence |  |  | -1466 | 2937 | 2953 | n.a. | n.a. |
| Self-Transcendence | linear |  | -1460 | 2928 | 2948 | 12 | <.01 |
| Self-Transcendence |  | Linear | -1444 | 2900 | 2931 | 31 | <.00 |
| Self-Transcendence | Squared | linear | -1443 | 2900 | 2936 | 3 | .11 |
| **Self-Transcendence** | **Squared, cubic** | **linear** | **-1441** | **2897** | **2938** | **5** | **.03** |
| Self-Transcendence | cubic | Linear, squared | -1438 | 2883 | 2903 | no con­ver­gence | no con |
| Self-Transcendence |  | Linear, squared, cubic | -1433 | 2895 | 2972 | 10 | .53 |

*Note*. All models include a random intercept term. Bold models are best fitting and used to create figures. n.a. = not applicable.

# LuNT Regression Tables

**Table H**

**Estimated Coefficients from Multilevel Growth Model of HOVs in the LuNT Dataset**

|  | Openness to change | Self-transcendence | Self-enhancement | Conservation |
| --- | --- | --- | --- | --- |
| Year | -.1581** | .047** | -.045* | .11** |
|  | (.0602) | (.018) | (.0205) | (.0182) |
|  | .0086 | .0089 | .0284 | 1.73e-09 |
| Year Squared | .0353** |  |  |  |
|  | (.0128) |  |  |  |
|  | .0058 |  |  |  |
| Intercept | 4.7552** | 6.19** | 4.13** | 4.67** |
|  | (.0889) | (.0712) | (.0812) | (.0766) |
|  | .0000 | 0 | 0 | 0 |
| S.D. Random Slope Year | .1604** | .176** | .181** | .161** |
|  | (.0309) | (.0203) | (.0261) | (.0237) |
|  | .0000 | 0 | 3.67e-12 | 1.10e-11 |
| S.D. Random Intercept | 1.0727** | .961** | 1.07** | 1.03** |
|  | (.0712) | (.0585) | (.0686) | (.063) |
|  | .0000 | 0 | 0 | 0 |
| Corr(I,S) | -.0609 | -.477** | -.252* | -.348** |
|  | (.1638) | (.0866) | (.122) | (.111) |
|  | .7101 | 3.63e-08 | .0397 | .0017 |
| Residual S.D. | .9114** | .699** | .85** | .753** |
|  | (.0236) | (.018) | (.0219) | (.0195) |
|  | .0000 | 0 | 0 | 0 |
| LL | -1892.7537 | -1601 | -1820 | -1686 |
| AIC | 3799.5073 | 3213 | 3651 | 3384 |
| BIC | 3835.1843 | 3244 | 3682 | 3415 |
| N | 1,208 | 1,209 | 1,206 | 1,207 |

*Note.* * p < .05 ** p < .01, S.E. in parenthesis, p-value below

# GSOEP Descriptive Statistics

**Table I**

**GSOEP Descriptive Statistics**

| Variable | Mean | SD | min | max | Observations |
| --- | --- | --- | --- | --- | --- |
|  |  |  |  |  |  |
| Gender | 1.550 | 0.500 | 1 | 2 | 19566 |
| [1] Male (%) | 44.99 |  |  |  | 8802 |
| [2] Female (%) | 55.01 |  |  |  | 10764 |
| Year of birth | 1971 | 3.630 | 1966 | 1978 | 19566 |
| Age | 36.35 | 8.240 | 14 | 50 | 19566 |
| Education | 3.720 | 1.480 | 0 | 6 | 19442 |
| [0] in school (%) | 1.550 |  |  |  | 302 |
| [1] inadequately (%) | 1.750 |  |  |  | 341 |
| [2] general elementary (%) | 11.10 |  |  |  | 2158 |
| [3] middle vocational (%) | 46.26 |  |  |  | 8994 |
| [4] vocational + *Abitur* (%) | 9.880 |  |  |  | 1920 |
| [5] higher vocational (%) | 7.120 |  |  |  | 1384 |
| [6] higher education (%) | 22.34 |  |  |  | 4343 |
| Tertiary Education Indicator | 0.340 | 0.470 | 0 | 1 | 19566 |
| Non-Tertiary Education (%) | 65.80 |  |  |  | 12874 |
| Tertiary Education (%) | 34.20 |  |  |  | 6692 |
| Survey year | 2007 | 7.900 | 1990 | 2016 | 19566 |
| Openness to change | 2.710 | 0.590 | 1 | 4 | 19566 |
| Conservation | 3.470 | 0.630 | 1 | 4 | 19566 |
| Self-enhancement | 2.990 | 0.520 | 1 | 4 | 19566 |
| Self-transcendence | 2.740 | 0.510 | 1 | 4 | 19566 |

| **Fig L**  **Sample Origin of Respondents in the GSOEP in Each Wave by Percentage and Frequency** |
| --- |
| 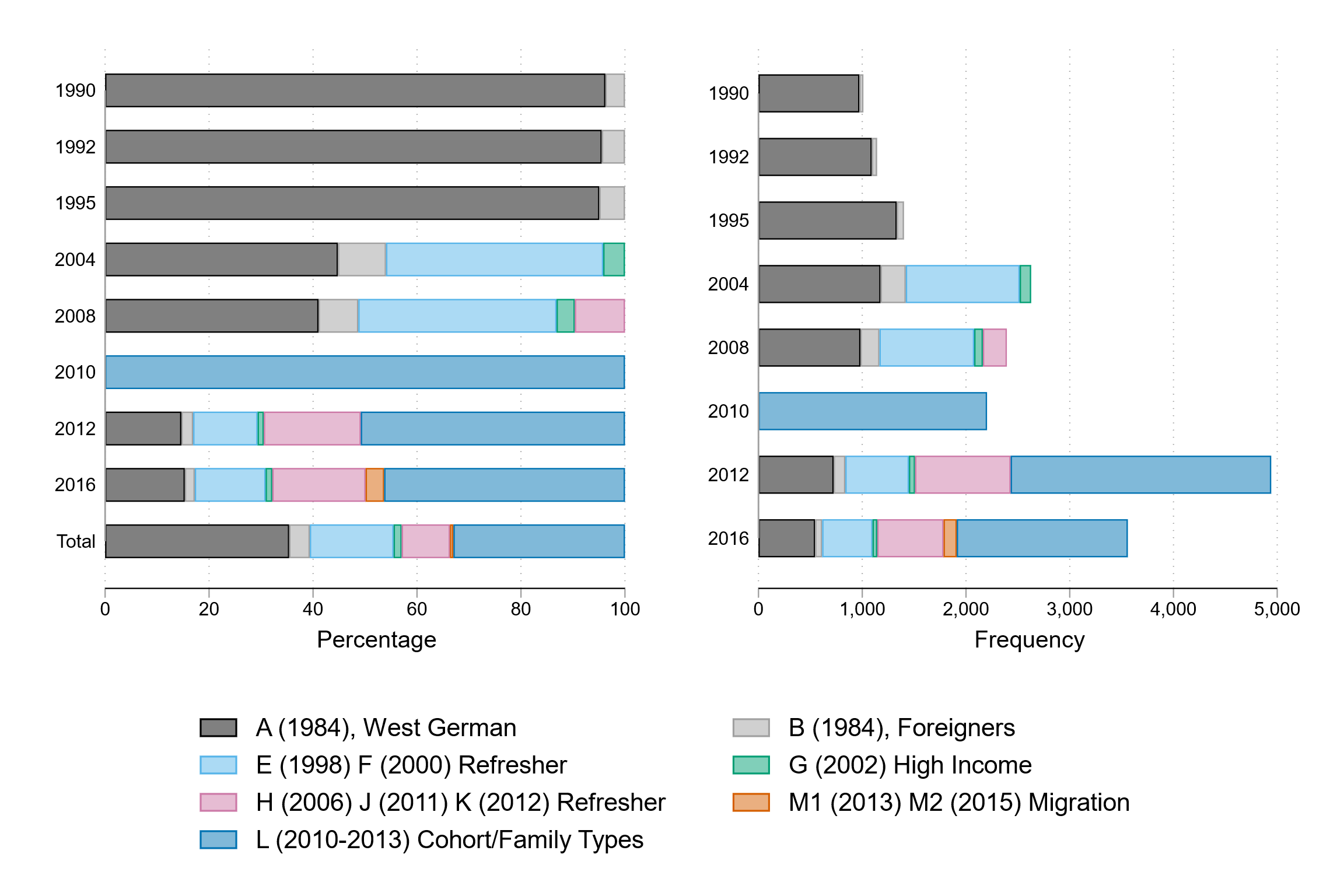 |

**Table J**

**Descriptive Statistics of HOV, Age, and Gender in Each GSOEP Wave**

| Year | Variable | Mean | SD | min | max | Observations |
| --- | --- | --- | --- | --- | --- | --- |
| 1990 | Conservation | 3.12 | 0.71 | 1.00 | 4.00 | 1011 |
| 1992 | Conservation | 3.17 | 0.71 | 1.00 | 4.00 | 1147 |
| 1995 | Conservation | 3.25 | 0.70 | 1.00 | 4.00 | 1445 |
| 2004 | Conservation | 3.43 | 0.64 | 1.00 | 4.00 | 2700 |
| 2008 | Conservation | 3.49 | 0.62 | 1.00 | 4.00 | 2457 |
| 2010 | Conservation | 3.72 | 0.42 | 1.50 | 4.00 | 2203 |
| 2012 | Conservation | 3.56 | 0.58 | 1.00 | 4.00 | 4999 |
| 2016 | Conservation | 3.52 | 0.60 | 1.00 | 4.00 | 3604 |
| 1990 | Openness to Change | 3.03 | 0.58 | 1.00 | 4.00 | 1011 |
| 1992 | Openness to Change | 2.98 | 0.59 | 1.00 | 4.00 | 1147 |
| 1995 | Openness to Change | 2.92 | 0.58 | 1.00 | 4.00 | 1445 |
| 2004 | Openness to Change | 2.75 | 0.58 | 1.00 | 4.00 | 2700 |
| 2008 | Openness to Change | 2.65 | 0.57 | 1.00 | 4.00 | 2457 |
| 2010 | Openness to Change | 2.60 | 0.58 | 1.00 | 4.00 | 2203 |
| 2012 | Openness to Change | 2.63 | 0.57 | 1.00 | 4.00 | 4999 |
| 2016 | Openness to Change | 2.62 | 0.57 | 1.00 | 4.00 | 3604 |
| 1990 | Self-enhancement | 2.59 | 0.52 | 1.00 | 4.00 | 1011 |
| 1992 | Self-enhancement | 2.56 | 0.53 | 1.00 | 4.00 | 1147 |
| 1995 | Self-enhancement | 2.58 | 0.50 | 1.00 | 4.00 | 1445 |
| 2004 | Self-enhancement | 2.66 | 0.49 | 1.00 | 4.00 | 2700 |
| 2008 | Self-enhancement | 2.60 | 0.49 | 1.00 | 4.00 | 2457 |
| 2010 | Self-enhancement | 2.96 | 0.48 | 1.50 | 4.00 | 2203 |
| 2012 | Self-enhancement | 2.81 | 0.49 | 1.00 | 4.00 | 4999 |
| 2016 | Self-enhancement | 2.82 | 0.50 | 1.00 | 4.00 | 3604 |
| 1990 | Self-transcendence | 3.21 | 0.52 | 1.00 | 4.00 | 1011 |
| 1992 | Self-transcendence | 3.20 | 0.51 | 1.00 | 4.00 | 1147 |
| 1995 | Self-transcendence | 3.18 | 0.51 | 1.50 | 4.00 | 1445 |
| 2004 | Self-transcendence | 3.11 | 0.51 | 1.00 | 4.00 | 2700 |
| 2008 | Self-transcendence | 3.03 | 0.49 | 1.50 | 4.00 | 2457 |
| 2010 | Self-transcendence | 2.85 | 0.53 | 1.00 | 4.00 | 2203 |
| 2012 | Self-transcendence | 2.93 | 0.51 | 1.00 | 4.00 | 4999 |
| 2016 | Self-transcendence | 2.85 | 0.51 | 1.00 | 4.00 | 3604 |
| 1990 | age | 20.92 | 2.26 | 14.00 | 24.00 | 1011 |
| 1992 | age | 22.22 | 2.82 | 16.00 | 26.00 | 1147 |
| 1995 | age | 24.17 | 3.77 | 17.00 | 29.00 | 1445 |
| 2004 | age | 33.01 | 3.72 | 26.00 | 38.00 | 2700 |
| 2008 | age | 37.04 | 3.70 | 30.00 | 42.00 | 2457 |
| 2010 | age | 38.21 | 3.65 | 32.00 | 44.00 | 2203 |
| 2012 | age | 40.80 | 3.65 | 34.00 | 46.00 | 4999 |
| 2016 | age | 44.77 | 3.66 | 38.00 | 50.00 | 3604 |
| 1990 | gender | 0.50 | 0.50 | 0.00 | 1.00 | 1011 |
| 1992 | gender | 0.50 | 0.50 | 0.00 | 1.00 | 1147 |
| 1995 | gender | 0.51 | 0.50 | 0.00 | 1.00 | 1445 |
| 2004 | gender | 0.52 | 0.50 | 0.00 | 1.00 | 2700 |
| 2008 | gender | 0.52 | 0.50 | 0.00 | 1.00 | 2457 |
| 2010 | gender | 0.60 | 0.49 | 0.00 | 1.00 | 2203 |
| 2012 | gender | 0.57 | 0.49 | 0.00 | 1.00 | 4999 |
| 2016 | gender | 0.57 | 0.50 | 0.00 | 1.00 | 3604 |

# Multilevel Growth Models of HOV from the GSOEP

All models follow the general formulation in Equation 1 which describes a multilevel model with several fixed predictors and a random intercept and random slope. In Equation 1, *i* refers to the *i^th^* observation of respondent *j*, the $\beta_{0}$ is the population intercept at year 1990, $\beta_{1}$ is the effect of one unit increase in time (year). The $\beta_{2}$ and $\beta_{3}$ are the squared and cubic effects of a one-unit increase in time, respectively. The $\beta_{4}$ is the effect of education attained at any point in time and $\beta_{5}$, $\beta_{6}$ and $\beta_{7}$ are the interaction effect of year with education. Lastly the term $u_{0j}$ refers to the random intercept and $u_{1j}year$ is the random effect of the variable year for each individual *j*, these terms allow individual variation in the intercept and effect of year.

$y_{ij}= \text{β}_{\text{0}}+\beta_{1}\mathrm{year}_{\mathrm{ij}}+\beta_{2}{\mathrm{year}^{2}}_{\mathrm{ij}}+\beta_{3}{\mathrm{year}^{3}}_{\mathrm{ij}}+\beta_{4}\mathrm{education}_{j}{+ \beta}_{5}{\mathrm{year}_{\mathrm{ij}}\mathrm{education}}_{j} {+ \beta}_{6}{{\mathrm{year}^{2}}_{\mathrm{ij}}\mathrm{education}}_{j} {+ \beta}_{7}{{\mathrm{year}^{3}}_{\mathrm{ij}}\mathrm{education}}_{j} +u_{0j}+u_{1j}\mathrm{year}$

**Table K**

**Description of Model Parameters**

| Model | Fixed effects | Random effects | Interaction effects |
| --- | --- | --- | --- |
| 1 | year | intercept |  |
| 2 |  | intercept, year |  |
| 3 | year^2^ | intercept, year |  |
| 4 | year^2^, year^3^ | intercept, year |  |
| 5 | year^2^, year^3^, education | intercept, year |  |
| 6 | year^2^, year^3^, education | intercept, year | education x year, education x year^2^, education x year^3^ |

**Table L**

**Likelihood Ratio Tests of Models Run on GSOEP Data**

| Higher Order Value | Model A | Model B | χ^2^ | df | *p* |
| --- | --- | --- | --- | --- | --- |
| Self-transcendence | 1 | 2 | 110.01 | 2.00 | <.01 |
|  | 2 | 3 | 0.01 | 1.00 | .94 |
|  | 3 | 4 | 42.42 | 1.00 | <.01 |
|  | 4 | 5 | 220.88 | 1.00 | <.01 |
|  | 5 | 6 | 3.31 | 3.00 | .35 |
| Openness | 1 | 2 | 170.85 | 2.00 | <.01 |
|  | 2 | 3 | 99.26 | 1.00 | <.01 |
|  | 3 | 4 | 11.41 | 1.00 | <.01 |
|  | 4 | 5 | 179.83 | 1.00 | <.01 |
|  | 5 | 6 | 1.52 | 3.00 | .68 |
| Conservation | 1 | 2 | 568.12 | 2.00 | <.01 |
|  | 2 | 3 | 275.97 | 1.00 | <.01 |
|  | 3 | 4 | 27.31 | 1.00 | <.01 |
|  | 4 | 5 | 15.02 | 1.00 | <.01 |
|  | 5 | 6 | 10.35 | 3.00 | .02 |
| Self-enhancement | 1 | 2 | 124.23 | 2.00 | <.01 |
|  | 2 | 3 | 11.78 | 1.00 | <.01 |
|  | 3 | 4 | 12.01 | 1.00 | <.01 |
|  | 4 | 5 | 8.78 | 1.00 | <.01 |
|  | 5 | 6 | 26.88 | 3.00 | <.01 |

**Table M**

**AIC and BIC Statistics from Multilevel Growth Models Using GSOEP**

| Outcome | Model | LL | AIC | BIC | N |
| --- | --- | --- | --- | --- | --- |
| conservation | 1 | -16189 | 32385 | 32417 | 19566 |
| conservation | 2 | -15905 | 31821 | 31868 | 19566 |
| conservation | 3 | -15767 | 31547 | 31602 | 19566 |
| conservation | 4 | -15753 | 31522 | 31585 | 19566 |
| conservation | 5 | -15745 | 31509 | 31580 | 19566 |
| **conservation** | **6** | **-15740** | **31505** | **31599** | **19566** |
| openness to change | 1 | -15064 | 30135 | 30167 | 19566 |
| openness to change | 2 | -14978 | 29969 | 30016 | 19566 |
| openness to change | 3 | -14929 | 29871 | 29926 | 19566 |
| openness to change | 4 | -14923 | 29862 | 29925 | 19566 |
| **openness to change** | **5** | **-14833** | **29684** | **29755** | **19566** |
| openness to change | 6 | -14832 | 29689 | 29783 | 19566 |
| self-enhancement | 1 | -13011 | 26029 | 26061 | 19566 |
| self-enhancement | 2 | -12948 | 25909 | 25956 | 19566 |
| self-enhancement | 3 | -12943 | 25899 | 25954 | 19566 |
| self-enhancement | 4 | -12937 | 25889 | 25952 | 19566 |
| self-enhancement | 5 | -12932 | 25882 | 25953 | 19566 |
| **self-enhancement** | **6** | **-12919** | **25861** | **25956** | **19566** |
| self-transcendence | 1 | -12809 | 25627 | 25659 | 19566 |
| self-transcendence | 2 | -12754 | 25521 | 25568 | 19566 |
| self-transcendence | 3 | -12754 | 25523 | 25578 | 19566 |
| self-transcendence | 4 | -12733 | 25483 | 25546 | 19566 |
| **self-transcendence** | **5** | **-12623** | **25264** | **25335** | **19566** |
| self-transcendence | 6 | -12621 | 25266 | 25361 | 19566 |

*Note*. Models in bold are best fitting and used to create figures.

# Standardized Estimated Mean Level and Slope of HOV in GSOEP

| **Fig M**  **Estimated Means of HOV (Standardized) in the GSOEP** |
| --- |
| 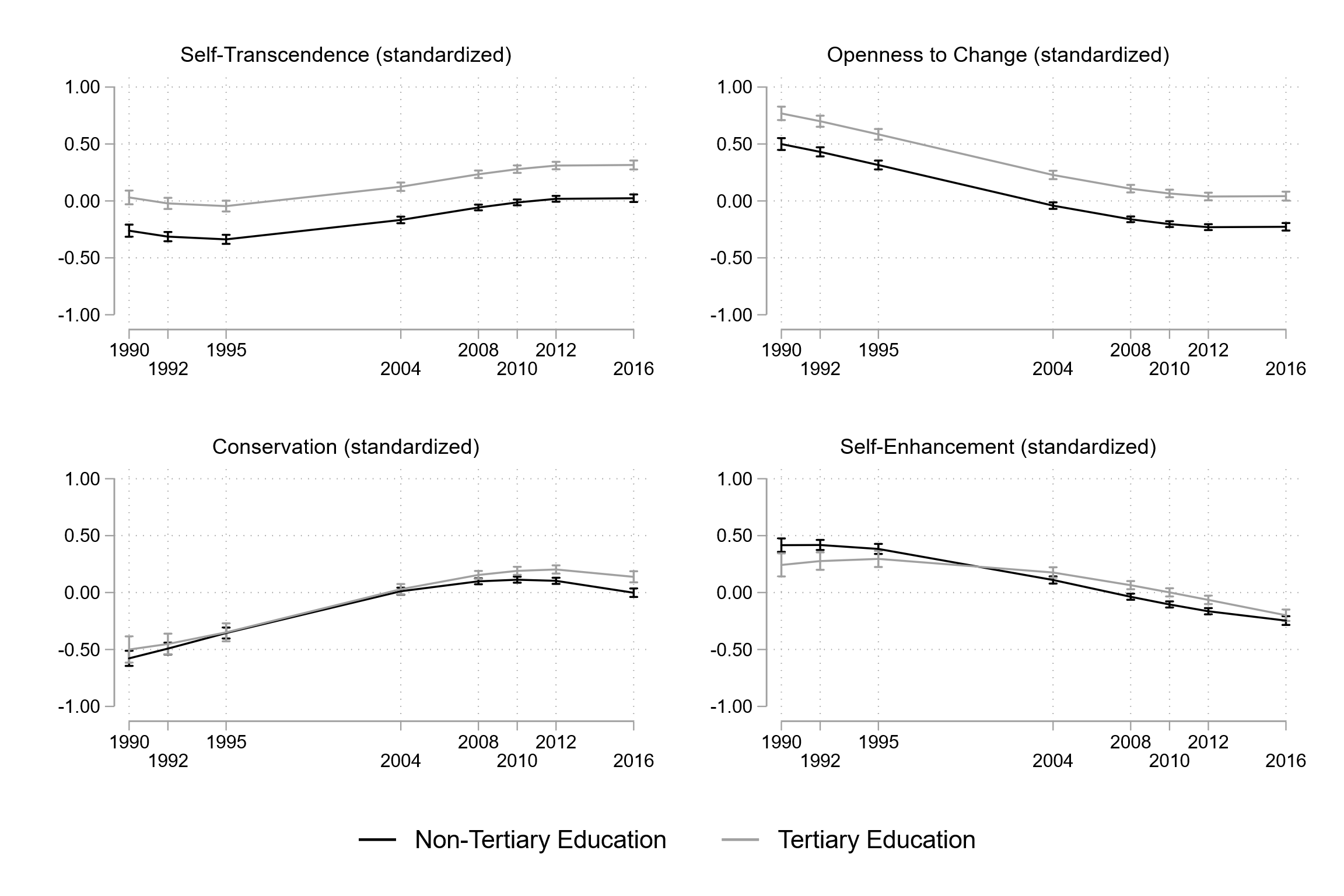 |

| **Fig N**  **Estimated Slopes of HOV (Standardized) in the GSOEP** |
| --- |
| 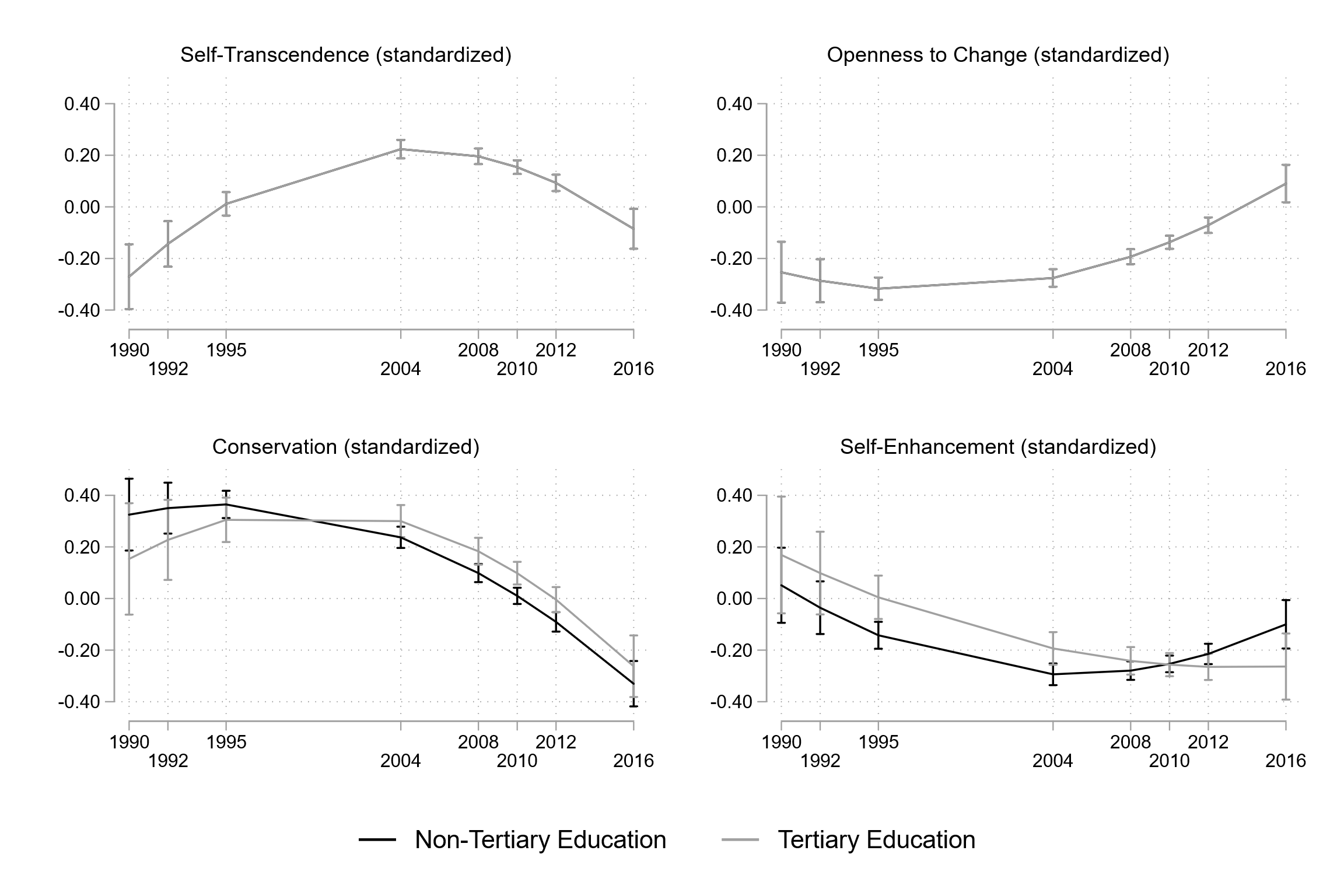 |

# Estimated Coefficients from Multilevel Growth Modelling of HOVs in the GSOEP Dataset

**Table N**

**Multilevel Growth Model of Conservation—Models 1 to 6 Using the GSOEP Data**

| Conservation | Model 1 | Model 2 | Model 3 | Model 4 | Model 5 | Model 6 |
| --- | --- | --- | --- | --- | --- | --- |
| Intercept | 3.249** | 3.265** | 3.091** | 3.123** | 3.112** | 3.111** |
|  | (.011) | (.014) | (.017) | (.018) | (.019) | (.021) |
| Higher Education |  |  |  |  | .048** | .049 |
|  |  |  |  |  | (.012) | (.043) |
| Year | .052** | .051** | .177** | .088** | .087** | .103** |
|  | (.002) | (.003) | (.008) | (.019) | (.019) | (.023) |
| Higher Education x Year |  |  |  |  |  | -.055 |
|  |  |  |  |  |  | (.042) |
| Year Squared |  |  | -.017** | .015* | .015* | .010 |
|  |  |  | (.001) | (.006) | (.006) | (.007) |
| Higher Education x Year Squared |  |  |  |  |  | .016 |
|  |  |  |  |  |  | (.013) |
| Year cubic |  |  |  | -.003** | -.003** | -.003** |
|  |  |  |  | (.001) | (.001) | (.001) |
| Higher Education x Year cubic |  |  |  |  |  | -.001 |
|  |  |  |  |  |  | (.001) |
| S.D. Random Slope Year |  | .104** | .103** | .103** | .103** | .103** |
|  |  | (.003) | (.003) | (.003) | (.003) | (.003) |
| S.D. Random Intercept | .423** | .599** | .590** | .589** | .589** | .588** |
|  | (.005) | (.014) | (.014) | (.014) | (.014) | (.014) |
| Corr (I,S) |  | -.730** | -.719** | -.720** | -.720** | -.719** |
|  |  | (.015) | (.016) | (.015) | (.016) | (.016) |
| Residual S.D. | .442** | .411** | .407** | .407** | .407** | .407** |
|  | (.003) | (.003) | (.003) | (.003) | (.003) | (.003) |
| LL | -16188.651 | -15904.590 | -15766.604 | -15752.948 | -15745.439 | -1574.264 |
| AIC | 32385.303 | 31821.180 | 31547.209 | 31521.896 | 31508.879 | 31504.527 |
| BIC | 32416.830 | 31868.469 | 31602.381 | 31584.949 | 31579.813 | 31599.105 |
| N | 19,566 | 19,566 | 19,566 | 19,566 | 19,566 | 19,566 |

**Table O**

**Multilevel Growth Model of Openness to Change—Models 1 to 6 Using the GSOEP Data**

| Openness to change | Model 1 | Model 2 | Model 3 | Model 4 | Model 5 | Model 6 |
| --- | --- | --- | --- | --- | --- | --- |
| Intercept | 2.989** | 2.980** | 3.061** | 3.040** | 3.002** | 2.999** |
|  | (.011) | (.012) | (.014) | (.015) | (.016) | (.018) |
| Higher Education |  |  |  |  | .159** | .168** |
|  |  |  |  |  | (.012) | (.035) |
| Year | -.064** | -.062** | -.130** | -.074** | -.076** | -.068** |
|  | (.002) | (.002) | (.007) | (.018) | (.018) | (.021) |
| Higher Education x Year |  |  |  |  |  | -.026 |
|  |  |  |  |  |  | (.040) |
| Year Squared |  |  | .010** | -.010 | -.011 | -.015* |
|  |  |  | (.001) | (.006) | (.006) | (.007) |
| Higher Education x Year Squared |  |  |  |  |  | .011 |
|  |  |  |  |  |  | (.013) |
| Year Cubic |  |  |  | .002** | .002** | .002** |
|  |  |  |  | (.001) | (.001) | (.001) |
| Higher Education x Year Cubic |  |  |  |  |  | -.001 |
|  |  |  |  |  |  | (.001) |
| S.D. Random Slope Year |  | .069** | .068** | .068** | .068** | .068** |
|  |  | (.003) | (.003) | (.003) | (.003) | (.003) |
| S.D. Random Intercept | .402** | .440** | .440** | .439** | .434** | .434** |
|  | (.005) | (.012) | (.012) | (.012) | (.012) | (.012) |
| Corr(I,S) |  | -.487** | -.482** | -.482** | -.494** | -.494** |
|  |  | (.034) | (.034) | (.034) | (.033) | (.033) |
| Residual S.D. | .416** | .403** | .401** | .401** | .401** | .401** |
|  | (.003) | (.003) | (.003) | (.003) | (.003) | (.003) |
| LL | -15063.710 | -14978.286 | -14928.656 | -14922.949 | -14833.036 | -14832.275 |
| AIC | 30135.420 | 29968.572 | 29871.313 | 29861.898 | 29684.072 | 29688.551 |
| BIC | 30166.945 | 30015.861 | 29926.484 | 29924.951 | 29755.006 | 29783.129 |
| N | 19,566 | 19,566 | 19,566 | 19,566 | 19,566 | 19,566 |

**Table P**

**Multilevel Growth Model of Self-enhancement—Models 1 to 6 Using the GSOEP Data**

| Self-enhancement | Model 1 | Model 2 | Model 3 | Model 4 | Model 5 | Model 6 |
| --- | --- | --- | --- | --- | --- | --- |
| Intercept | 3.225** | 3.233** | 3.209** | 3.189** | 3.182** | 3.211** |
|  | (.010) | (.010) | (.012) | (.014) | (.014) | (.016) |
| Higher Education x Intercept |  |  |  |  | .031** | -.091** |
|  |  |  |  |  | (.010) | (.031) |
| Year | -.053** | -.054** | -.033** | .019 | .019 | .014 |
|  | (.002) | (.002) | (.006) | (.017) | (.017) | (.020) |
| Higher Education |  |  |  |  |  | .031 |
|  |  |  |  |  |  | (.036) |
| Year Squared |  |  | -.003** | -.022** | -.022** | -.025** |
|  |  |  | (.001) | (.006) | (.006) | (.007) |
| Higher Education x Year Squared |  |  |  |  |  | .005 |
|  |  |  |  |  |  | (.012) |
| Year Cubic |  |  |  | .002** | .002** | .002** |
|  |  |  |  | (.001) | (.001) | (.001) |
| Higher Education x Year Cubic |  |  |  |  |  | -.001 |
|  |  |  |  |  |  | (.001) |
| S.D. Random Slope Year |  | .058** | .057** | .058** | .058** | .057** |
|  |  | (.003) | (.003) | (.003) | (.003) | (.003) |
| S.D. Random Intercept | .340** | .362** | .362** | .361** | .362** | .360** |
|  | (.004) | (.011) | (.011) | (.011) | (.011) | (.011) |
| Corr (I,S) |  | -.464** | -.462** | -.464** | -.470** | -.457** |
|  |  | (.040) | (.040) | (.040) | (.039) | (.041) |
| Residual S.D. | .383** | .373** | .373** | .373** | .373** | .373** |
|  | (.002) | (.003) | (.003) | (.003) | (.003) | (.003) |
| LL | -1301.578 | -12948.462 | -12942.571 | -12936.565 | -12932.175 | -12918.735 |
| AIC | 26029.156 | 25908.924 | 25899.143 | 25889.131 | 25882.350 | 25861.471 |
| BIC | 2606.682 | 25956.213 | 25954.314 | 25952.184 | 25953.283 | 25956.049 |
| N | 19,566 | 19,566 | 19,566 | 19,566 | 19,566 | 19,566 |

**Table Q**

**Multilevel Growth Model of Self-Transcendence—Models 1 to 6 Using the GSOEP Data**

| Self-transcendence | Model 1 | Model 2 | Model 3 | Model 4 | Model 5 | Model 6 |
| --- | --- | --- | --- | --- | --- | --- |
| Intercept | 2.621** | 2.605** | 2.604** | 2.640** | 2.604** | 2.591** |
|  | (.010) | (.010) | (.013) | (.014) | (.014) | (.016) |
| Higher Education |  |  |  |  | .149** | .201** |
|  |  |  |  |  | (.010) | (.032) |
| Year | .030** | .033** | .033** | -.066** | -.070** | -.062** |
|  | (.002) | (.002) | (.006) | (.017) | (.017) | (.020) |
| Higher Education x Year |  |  |  |  |  | -.031 |
|  |  |  |  |  |  | (.036) |
| Year Squared |  |  | -.000 | .035** | .035** | .034** |
|  |  |  | (.001) | (.006) | (.005) | (.007) |
| Higher Education x Year Squared |  |  |  |  |  | .005 |
|  |  |  |  |  |  | (.012) |
| Year Cubic |  |  |  | -.003** | -.003** | -.003** |
|  |  |  |  | (.001) | (.001) | (.001) |
| Higher Education x Year Cubic |  |  |  |  |  | -.000 |
|  |  |  |  |  |  | (.001) |
| S.D. Random Slope Year |  | .056** | .056** | .057** | .056** | .056** |
|  |  | (.003) | (.003) | (.003) | (.003) | (.003) |
| S.D. Random Intercept | .329** | .370** | .370** | .369** | .361** | .361** |
|  | (.004) | (.011) | (.011) | (.011) | (.011) | (.011) |
| Corr (I,S) |  | -.520** | -.520** | -.525** | -.527** | -.525** |
|  |  | (.035) | (.035) | (.035) | (.035) | (.035) |
| Residual S.D. | .382** | .373** | .373** | .373** | .373** | .373** |
|  | (.002) | (.003) | (.003) | (.003) | (.003) | (.003) |
| LL | -12809.495 | -12754.487 | -12754.484 | -12733.274 | -12622.836 | -12621.182 |
| AIC | 25626.990 | 25520.975 | 25522.969 | 25482.549 | 25263.672 | 25266.363 |
| BIC | 25658.516 | 25568.264 | 25578.139 | 25545.602 | 25334.605 | 25360.941 |
| N | 19,566 | 19,566 | 19,566 | 19,566 | 19,566 | 19,566 |
