# Supplementary material for: Are values stable throughout adulthood? Evidence from two German long-term panel studies
Source: PLoS One. 2023 Nov 30;18(11):e0289487. doi: 10.1371/journal.pone.0289487 (PMC10688669; doi:10.1371/journal.pone.0289487)
Supplement: Attachment — Submitted filename: Planned responses (1).docx [file pone.0289487.s002.docx]

# Editor

Please include the following items when submitting your revised manuscript:

- A rebuttal letter that responds to each point raised by the academic editor and reviewer(s). You should upload this letter as a separate file labeled 'Response to Reviewers'.
- A marked-up copy of your manuscript that highlights changes made to the original version. You should upload this as a separate file labeled 'Revised Manuscript with Track Changes'.
- An unmarked version of your revised paper without tracked changes. You should upload this as a separate file labeled 'Manuscript'.

# Reviewer #1: Review PLOS ONE – PONE-D-23-01126

This research contributes to the literature, but the claim that this is the first study “to systematically investigate whether values remain stable across the lifespan or change throughout adulthood” (p. 4) and on p. 10 (first sentence under The Present Research”) is not warranted. The article by Leijen, Van Herk and Bardi (2022) in Scientific Reports covers the same topic using a large representative sample from the Netherlands, covering 12 years (2008-2020) and includes the same individuals who were between 16 and 84 (in 2008) and were present in all 7 time-points. This article should be incorporated into the introduction and its results should be reflected upon in the results and discussion sections of the current manuscript. The Leijen et al. (2022) article has similarities to the current manuscript, and it is insightful and important to reflect on these outcomes from Germany's neighboring country in the current manuscript. It should be noted, however, that the approach taken by the current manuscript with a focus on years is different from Leijen et al. (2022) and provides novel insight.

- Citation included.
- We also updated the text at various places and highlighted the Leijen et al. study contribution.
- We also made sure to no longer refer to our study as the first that examined value development in a true longitudinal approach.

On page 6, some references to current work on value change in children could be cited (e.g., Daniel et al., 2020).

- Reference to Daniel et al., 2020 and Daniel et al. 2023 now included at p. 6.

On p. 7 the article by Leijen et al. (2022) could be included to emphasize the point that longitudinal studies indicate values change in individuals aged 16-84.

- At pp. 7-8, we now summaries the results of Leijen et al. (2022). Also see quote:
  “Leijen et al. (2022) studied changes in values among Dutch individuals aged 16 to 84 over a 12-year period and across four generations. The study found that changes within individuals occurred in all generations, but were more significant in the younger respondents, the Millennials. Millennials increased the importance of benevolence, universalism, self-direction and security while stimulation and power decreased with time. The values of hedonism, achievement and conformity did not change within individuals. This study is unique in its approach, as it is the only one using longitudinal data longer than three years to observe value change in adulthood. However, the authors cautioned against generalizing their findings to other cultural and socio-economic contexts, raising the question of whether the observed value development in adulthood is unique to the Dutch population or has universal significance.” (pp.7-8)

On page 11, the part on GSOEP needs elaboration. It is not clear from the text how old the individuals were in the 26 years mentioned. Please clarify.

- Similar to the presentation of LuNT study, we now provide the age at the first round of GSOEP and the year-range when the study participants were born.

“In the first wave in 1990, participants were on average 21 years old (born between 1966 and 1976).” (p.11)

At the end of page 12 the sample description of Study 1 is unclear. It is secondary data, so it seems to me that the authors did not make decisions on sampling. Who did and where is it reported. Please elaborate this part of the text.

- The information previously presented at the end of page 12 describes the sampling strategy of Study 1, which is the study we carried out to ensure comparability of measurement used in the LuNT and GSOEP panels. Study 1 therefore does not rely on secondary but primary data. This was stated previously in the following sentence, which we now clarify further:

“We collected data in an online panel for Study 1 to create a covariance matrix of all items used in Study 2 and Study 3, which both rely on secondary data sources.” (p.13)

At the end of page 14, it is stated that missing on more than 75% of the items was seen as problematic. Does this mean that 74% or less missing is considered fine? When that was the case, what was done? Imputing? How? What is the distribution of missing across individuals? Please clarify.

- In line with recommendations in the literature (Schwartz, 2003), we expected that missing information on more than 75% of the items of an instrument is insufficient for purposes of deriving reliable and valid higher order value measures. Schwartz further recommends to drop cases which have the same response on more than 75% of items. In the present research (Study 1) we therefore had to drop 3 cases that responded in the same manner to more than 75% of items on all three of the value instruments. We did not drop cases for missing values as reported earlier.

“For quality control, cases with missing information on more than 75% of the items of an instrument or cases using the same response to at least 75% of items within an instrument, across all three instruments were dropped from analyses (also see Schwartz, 2003). In total, three cases were dropped due to response pattern, reducing the final sample size to *N* = 1,027.”

Page 17. The citation to the smacof package paper is outdated. The recent citation is Mair, Groenen, De Leeuw, 2022. This refers to the current updated package in R. See below.

- We understand the recommendation, but the reference is to the version we used therefore we will rather keep it.

p. 21 Is the “Longitudinal Internet Studies for the Social Sciences” the LISS panel?

- Indeed, this is the LISS panel. We added the acronym; please see at p. 21.

p. 30. The last sentence is unclear: “there is no sample is assessed in each measurement wave”. Please change.

- We cannot find the quote on page 30. However, a similar sentence appears on page 32.
- Pg 32: “There is no *core sample* that has been assessed in each and every measurement wave.” Could be changed to “There are no respondents that are observed over the entire period”.

On page 31 it is written that the GSOEP is a chain of shorter panels. This is fine but it also indicates a difference with the Leijen et al. (2022) study in which the same people were included in all waves over the 12-year period. That the results based on GSOEP are in line with the findings in the Netherlands again provides evidence for the way in which values change in adulthood. Also, shorter panels provide similar results.

- We have clarified the composition of the GSOEP with reference to the sample distribution figure in the appendix. Indeed, as the reviewer notes there seems to be some similarity between shorter and longer panel studies. However, GSOEP does also include participants who responded from 1990 until 2016 although they are a minority.
- “The GSOEP periodically draws refreshment samples (Infratest Sozialforschung, 2013) to decrease statistical biases due to dropout and non-response. In contrast to the LuNT data, the GSOEP contains respondents that were not observed over the entire study but still provides data spanning 26 years. The two main samples are the West German sample which includes respondents who answered in each wave from 1990 to 2016, except 2010, and the Cohort/Family types sample which started in 2010. However, most respondents in the sample provide data either from 1990 to 2008 or from 2010 onwards (N = 6103, 78%) while 22% of (N=1730) respondents provide data spanning the entire period from 1990 to 2016. For more information see Figure S9.1 in the appendix.”(p.29)

On page 37 at the end, I think a possible explanation for the return to a positive rate of change may be in the return of economic growth after the recession caused by the global financial crises (GFC) in 2008. People in their early 30s were then more affected by the GFC than older generations (e.g., through temporary unemployment or difficulty in getting a job) but were able to pick up quickly after the GFC.

- Thank you for pointing this out. We address as such in the general discussion:

“Across two different samples from Germany, we showed that value developmental trajectories in adulthood are not always linear therefore providing contradictory evidence from cross-sectional (e.g., Dobewall et al., 2017) and short-term longitudinal studies (e.g., Milfont et al., 2016). We observed a decline in the importance of openness-to-change in both the highly educated peace activist sample (Study 2) and the general population sample (Study 3) until approximately the age of 30. This occurred in the context of the socio-economic crisis of 2007-2008, during which younger generations faced high levels of unemployment and economic instability, which may have hindered their ability to pursue a lifestyle that involves stimulating activities and independent thinking. However, while the peace activist sample eventually returned to positive rates of change emerging out of the crisis, the general population sample plateaued thereafter. The plateau in the general population coincides with patterns found in the general population in New Zealand (Milfont et al., 2016) and the Netherlands (Leijen et al., 2022). There appears however to be something unique to the peace activists in Germany that made them experience differently the years after the crisis, and education is most likely not the answer. Perhaps their worries, or better yet the ability to find meaning in life despite having to cope with worries about an uncertain future might provide a better explanation (e.g., Ojala, 2005). In short, these discrepencies highlight the possibility that results in both samples reflect the impact of the life-span as well as historical events. ” (pp. 40-41)

Page 38. Change in HOV stabilizes in the late 40s. This may be related to Leijen et al. (2022) who find that values of Baby Boomers and the Silent generation hardly change over a 12-years period. Please reflect on this.

- We referred to this observation in our discussion of openness-to-change trends (see answer above and at pp. 40-41). See answer above.

# Reviewer #2:

**The paper is currently quite repetitive and long-winded.**

The measures and their histories are described repeatedly, the lack of longer-term longitudinal studies is stressed at length at various points, etc.

- We have cut and rewritten several parts of the introduction of the paper as well as the introductions of study 1, 2, and 3 to shorten the text.

At the same time, alternative models such as biological maturation, life history/life pace effects or normative pressures as well as contextual (in particular economic) changes are not mentioned (see for example Fischer, 2017 for a broad picture overview).

- We have added these possibilities in the general discussion.

The two longitudinal studies also overlap substantially and **constant backreferencing is occurring in study 3.** Possibly, the two longitudinal studies could be integrated into a single study?

- We have gone through Study 3 and focused on reporting results, any comparisons between S2 and S3 are now made in the general discussion section.

It may make sense to discuss the value model in terms of person vs social focused and anxiety vs growth values. This helps to map better onto sociological theories of values and it also may make it easier to explain some patterns.

Data analysis points:
For the validation study:
a) Please use raw data for all analyses and measures (at least check whether this makes a difference)

b) Probably a better approach would be to randomly split the sample, run a mds with all items together (the pvq and svs-10 items could be set to the theoretically expected position as per bilsky et al., the K&S items could be left free to vary), then use the second set to confirm the structure. The three analyses are all conducted on the same dataset and the final analysis is not independent from the first explorations.

- We have redone Study 1 in the manuscript following the suggestions of R2. Now it uses a split sample to explore data. The first split sample is analyzed using MDS based on correlation of all items (raw data ratings) and plots them based on the best configuration from 1000 random starts. The second split of the sample is a confirmatory analysis of the HOVs from SVS-10 and K-S and PVQ21 items. The analysis comes to largely the same conclusions except that conservation items of the K-S questionnaire in the exploratory MDS are closer to self-enhancement items than expected and occupy their own area. However, in the confirmatory MDS all HOV are close to their respective PVQ21 items. The K-S results are slightly different in that the conservation items “happy partnership” and “having children” are somewhere in between conservation and self-enhancement items. The item “help others” is closer to conservation but still near the transcendence quadrant. SVS-10: No differences between exploratory MDS with Ipsatized or raw items.

c) I am not sure whether this analysis shows measurement equivalence, it seems to show convergent validity for these measures in my books.

- We agree with R2 and have updated the text and refer now to construct and convergent validity instead of measurement invariance.

For both longitudinal studies:
How much variance in the variability is explained by these temporal effects? You could break down explained variance using the r2mlm package.

- In the GSOEP sample, the random slopes seem to have almost equal explained variance in openness to change, self-transcendence and self-enhancement values. In the conservation values temporal changes explain 10% of variance, double that of other variables. In all HOV, the largest percentage of explained variance is due to the random intercept.
- In the LuNT panel random intercepts explain almost 60% of variance while the ranom slopes vary between 2.8 and 5.5%. The explained variance of the openness to change slope is very small while the others are around 4 to 5%.
- Comparing the two studies, the GSOEP slopes explain much more variance than in the LuNT while in the LuNT study most variance is captured by the random intercepts.
- The estimates of the explained variance of the random slopes have been added to the results section covering the ICC in their respective studies.

| GSOEP Explained variance decomposition | | | | |
| --- | --- | --- | --- | --- |
| Dependent variable | Fixed Effect | Random Intercept | Random Slope | Total |
| Openness to change | 6.6% | 42.4% | 5.1% | 54.2% |
| Self-transcendence | 3.9% | 37.6% | 4.7% | 46.2% |
| Conservation | 5.0% | 42.5% | 10.5% | 58.0% |
| Self-enhancement | 4.4% | 40.0% | 4.6% | 49.0% |

| LuNT Explained variance decomposition | | | | |
| --- | --- | --- | --- | --- |
| Dependent variable | Fixed Effect | Random Intercept | Random Slope | Total |
| Openness to change | 0.3% | 57.7% | 2.8% | 60.8% |
| Self-transcendence | 0.5% | 56.1% | 5.5% | 62.1% |
| Conservation | 1.8% | 58.9% | 3.8% | 64.5% |
| Self-enhancement | 0.3% | 57.5% | 4.0% | 61.8% |

Study 3 – why not use the full data set? This would be much stronger than trying to match to the not representative sample of peace activists. I strongly recommend repeating the analyses with the full dataset.

- A study using the whole GSOEP sample would no doubt be very valuable to the literature. However, this paper focuses on leveraging the unique LuNT dataset. Our principle aim with the GSOEP was to examine to what extent the peace activists’ data differed from a more general sample of the population. In short, by using the GSOEP sample to mirror the LuNT we are able to compare the value trajectories of the peace activists with a similar more general sample and from there draw some lessons about the impact of the environment and the life-course. Thus, we feel that taking the whole sample would require us to rewrite the paper. In particular, using the whole sample of the GSOEP would require us to pay substantial attention to the migration histories and patterns between East and West Germany, emigration to Germany and cohort changes.

How did you deal with possible changes in education during this period (e.g., evening school, late entry to university)?

- We created non-time varying education indicator variables that correspond to the highest level of education observed during the entire course of data gathering. We only considered broad formal qualifications such as primary, secondary, and tertiary educational diplomas. German education is highly regulated, and it is unlikely that individuals will pursue some education that does not lead to a formally recognized diploma as these are often pre-requisites for jobs and are also seen as legitimate signals of skills by employers – who are often involved in creating the curriculum at least in the vocational level.

Data interpretation question:
To what extent could these effects be cohort or event effects? For example, for the peace activist cohort: Could it be that 2009 was particularly depressing economically (remember that it was around the world economic crisis and I think Germany suffered significantly around that time) and therefore resulted in lower openness, which then rebounded 10 years later? This also seems to fit the increasing conservatism (security?) concern over time. Or are there some other external factors that could influence these value scores? The Gaza war? The continuing casualties in Iraq? Somewhat similar patterns seem to appear in the representative sample. I am just trying to highlight that the analysis is not free from external forces that may influence value shifts.

- Thank you for the comments. The separation cohort-age-period effects is always difficult. With the simple models in the paper and without more data it is not possible to distinguish the time trends from cohort and period effects.
- We have added to the limitations section a sentence “For example, the impact of some event like the financial crises or the war in Iraq may have affected the LuNT participants differently than the general population, causing the discrepancies in value development observed in openness to change values.” We have also added the possibility of the impact of the 2008 financial crisis when comparing the results from the two samples in the general discussion.
- We have cut the paragraph that unambiguously attributes changes observed in the paper to life-span development in the general discussion and included (historical) events as a possible cause in the limitations and future research section. Cut Paragraph: “It furthermore seems that the way (western) societies are normatively structured along pre-defined and age-appropriate events, such as completing education, finding a job, starting a family, retiring, and eventually preparing for death is being reflected in the values of people (Witte, Stanciu, & Boehnke, 2020). Our analyses reveal an additional insight, namely the relevance of education for value development. We found that the level of education (and possibly the associated life events) can have an impact on the trajectories of individual developmental curves.

For study 3: The effect of education on valuing conservation values more in mid-life makes sense evolutionary speaking. For example, at this moment in life more highly educated individuals are entering positions of power within a social hierarchy, so they have to benefit more from upholding and preserving the system (I think some of the work within social dominance theory and biological theories of social status could be useful).

- Admittedly, we are not great friends of evolutionary psychology and/or evolutionary sociology. These conceptual frames claim to be able to explain the world based on alleged evidence from tens of thousands of years. The evidence that is brought forward is, however, not beyond doubt (e.g., with regard to gender relationships) and variables like chronological age have changed their meaning drastically since the neolithic era (due to dramatically different life expectancies). We, thus, prefer to just briefly mention evolutionary considerations as a possible explanation. “One interpretation of this finding is that individuals who invest economically in a social system and derive social status from it will also defend and conform to it (Fischer, 2018).” (p. 38)

Minor points:
The Milfont et al study is problematic in that they seem to find negative slopes for all values, suggesting that all values decreased over their 3 year study period. I found this hard to interpret (beyond some measurement bias effect) and if I am not mistaken, the authors did not attempt to provide an explanation either (I could misremember).

- We can confirm this happens as well with the LISS panel in the NL when looking at ratings of items. It seems that mean ratings drop over time. We have also been puzzled by the findings but believe it is a measurement issue where respondents re-center on the scale over time. We have this hunch because the ipsatized ratings show both positive and negative rates of change in line with other studies. Also, there were some studies showing that reflecting on values changes values as they are mostly internalized through non-explicit processes and that changing values which have an explicit reasoning is more difficult (by Maio if we recall correctly). Therefore, doing a values questionnaire may lead respondents to reflect more closely on values and reassess.
- In the LuNT and GSOEP we do find positive slopes. Perhaps it has to do with the different scales, in LuNT the scale goes from -1 “against my values” to 7, which may prevent respondents from recentering lower on the scale over time. In GSOEP it is a scale without a middle point where the labels on the left are rejections rather than labels like “not very like me” as in the PVQ used in Milfont et al.

Smacof not smacoff

- Corrected to smacof.
